# Maternal Metabolic Profiles, Their Relationships with Progesterone and Pregnancy-Associated Glycoprotein (PAG), and Biomarker Potential During Early Pregnancy in Lactating Ewes

**DOI:** 10.3390/vetsci13101003

**Published:** 2026-09-23

**Authors:** Gökhan Uyanık, Ahmet Gözer, Murat Abay, Ufuk Kaya, Ishak Gökcek, Ramazan Sertkol

**Affiliations:** 1 Department of Obstetrics and Gynaecology, Faculty of Veterinary Medicine, Hatay Mustafa Kemal University, 31060 Hatay, Türkiye; ahmetgozer@mku.edu.tr (A.G.); vetramazan8589@gmail.com (R.S.); 2 Department of Obstetrics and Gynaecology, Faculty of Veterinary Medicine, Erciyes University, 38280 Kayseri, Türkiye; mabay@erciyes.edu.tr; 3 Department of Biostatistics, Faculty of Veterinary Medicine, Hatay Mustafa Kemal University, 31060 Hatay, Türkiye; 4 Department of Physiology, Faculty of Veterinary Medicine, Hatay Mustafa Kemal University, 31060 Hatay, Türkiye; igokcek@mku.edu.tr

**Keywords:** ewes, pregnancy, metabolic profile, progesterone, pregnancy-associated glycoproteins, oxidative stress

## Abstract

This study aimed to characterize the longitudinal maternal biochemical phenotype associated with early pregnancy, determine its relationships with progesterone (P_4_) and pregnancy-associated glycoproteins (PAGs), and conduct an exploratory evaluation of the prognostic and discriminatory potential of selected variables. Following synchronized breeding outside the breeding season, serum samples were collected from lactating ewes with confirmed singleton pregnancies (*n* = 22) and non-pregnant ewes (*n* = 22) at six time points from mating to D50. Pregnant ewes showed lower total protein and globulin, higher albumin-to-globulin ratio (A/G), and higher lactate dehydrogenase (LDH) from D7 to D35. Total oxidant status (TOS) was lower at mating and D14 in ewes that subsequently became pregnant. In pooled analyses, the P_4_-PAG correlation increased from D21 (r = 0.353) to D50 (r = 0.844), whereas most biochemical associations were attenuated in pregnancy-only analyses, suggesting that some pooled relationships were partly influenced by pregnancy-status separation. TOS showed the highest apparent prognostic performance at mating (AUC = 0.939). On D7, P_4_ (AUC = 0.948) and LDH (AUC = 0.915) showed the highest apparent individual discriminatory performance. By D21, P_4_ provided complete in-sample discrimination (AUC = 1.000), while A/G was the highest apparent performance among non-hormonal variables (AUC = 0.916). Exploratory multivariable models indicated the greatest apparent potential for biochemical–endocrine complementarity during D7-D14. These findings support a dynamic maternal biochemical phenotype and identify routinely measurable candidate variables that may complement luteal and conceptus–placental indicators, although these performance estimates are hypothesis-generating and require external validation.

## Simple Summary

Early pregnancy is a critical period for reproductive efficiency in ewes, but the systemic changes associated with successful pregnancy establishment before conventional diagnosis remain incompletely understood. This study evaluated whether routine blood measurements could characterize these changes and support earlier reproductive assessment. Pregnant ewes were compared with non-pregnant ewes from mating to day 50. Pregnant ewes showed coordinated changes in serum proteins, lipids, minerals, hormones, and the balance between oxidants and protective antioxidants. Some differences were already evident at mating and therefore likely reflected the pre-existing physiological status of the ewes, whereas others developed after mating as pregnancy establishment progressed. Several routinely measurable blood variables also showed potential for predicting subsequent pregnancy outcomes during the first two weeks after mating. These variables provided information complementary to that obtained from progesterone, a hormone reflecting ovarian luteal function that supports pregnancy, and pregnancy-associated glycoproteins, substances released by placental cells. Integrating routine biochemical measurements with ovarian and placental indicators may help veterinarians assess pregnancy outcome earlier, support timely rebreeding and nutritional management, and improve reproductive efficiency in sheep flocks.

## 1. Introduction

The establishment of pregnancy in ewes is characterized by coordinated maternal adaptations that begin immediately after mating and rapidly develop during the first weeks of gestation [1,2]. During this period, the conceptus undergoes rapid proliferation and elongation, establishes intensive molecular communication with the endometrium, generates signals required for maternal recognition of pregnancy, and supports the maintenance of luteal function [1,3]. Successful progression of these events requires the maternal organism to adapt to the changing nutritional, endocrine, and cellular demands of the developing conceptus [4].

Early conceptus development requires a continuous supply of substrates for energy production, cellular proliferation, membrane and protein synthesis, steroidogenesis, and tissue differentiation [5,6,7]. Meeting these requirements involves reorganization of maternal nutrient utilization and partitioning. Protein and nitrogen metabolism contribute to the availability and distribution of amino acids and other nitrogenous substrates between maternal and conceptus tissues [7,8,9], whereas lipid and lipoprotein metabolism supports energy transport, membrane synthesis, and the supply of lipid and cholesterol substrates required for steroidogenesis [10,11]. Mineral metabolism also supports fundamental processes including adenosine triphosphate (ATP) production, phosphorylation, cellular signaling, and tissue development [12,13,14]. These metabolic adaptations must occur within an appropriately regulated redox environment [15]. Reactive oxygen species contribute physiologically to steroidogenesis, cellular signaling, vascular adaptation, and tissue remodeling [16,17], whereas excessive oxidant production may adversely affect luteal function, endometrial homeostasis, and embryonic development [18,19,20]. Early pregnancy should therefore be considered not only a period of local reproductive events, but also a systemic maternal transition involving coordinated metabolic, endocrine, mineral, and redox adaptations.

However, the extent to which this physiological reorganization is reflected in the maternal circulation, and its temporal pattern during early pregnancy, remains insufficiently characterized in ewes. Much of the available evidence derives from studies of the endometrium, uterine lumen, conceptus, or placenta, which have demonstrated substantial changes in nutrient transport, energy metabolism, lipid composition, and molecular signaling during conceptus elongation and early placental development [4,8,11,21]. In contrast, less is known about when and to what extent these local adaptations are reflected in routinely measurable maternal blood variables. Most studies evaluating circulating biochemical profiles in ewes have focused on mid- or late gestation, maternal undernutrition, or metabolic disorders such as pregnancy toxemia [15,22,23]. More recently, metabolomic and proteomic studies have shown that pregnancy generates measurable systemic signatures in maternal circulation [24,25,26]. Nevertheless, it remains unclear to what extent these omics-derived changes can be represented by routinely measurable protein, lipid, energy, mineral, and oxidative variables, and how early such differences emerge.

Beyond defining the maternal biochemical phenotype, it is also important to determine how this phenotype relates to key reproductive processes involved in pregnancy progression. Progesterone (P_4_) and pregnancy-associated glycoproteins (PAGs) represent two established indicators reflecting biologically distinct aspects of early pregnancy physiology [27]. P_4_ primarily reflects corpus luteum (CL) steroidogenic activity and the luteal endocrine environment required for uterine receptivity, conceptus development, and pregnancy maintenance [28], whereas PAGs are produced by trophoblast-derived placental cells and provide more direct information on conceptus–placental development [29,30,31]. Their temporal profiles also differ: luteal P_4_ provides information on CL function shortly after mating [27], whereas circulating PAG concentrations progressively increase as trophoblast differentiation and placental development advance [29]. Accordingly, evaluating the maternal biochemical profile together with P_4_ and PAG may help define the temporal relationships between systemic metabolic changes and luteal and conceptus–placental activity, and provide a more integrated view of the maternal physiological response during early pregnancy.

Establishing these biological relationships may also clarify the potential value of maternal biochemical variables for early pregnancy assessment. This is particularly relevant during the early post-mating period when ultrasonographic findings may still be limited [32]. A longitudinal approach may further distinguish prognostic features observed before maternal recognition of pregnancy from candidate discriminatory biomarkers that emerge as pregnancy becomes established. Indeed, omics-based studies have shown that multivariate circulating signatures can successfully classify pregnancy status in ewes [24,25,26]. However, an important gap remains between these discovery-oriented approaches and biomarker strategies applicable in routine laboratory settings. To the best of our knowledge, no study has comprehensively evaluated, within the same population, the longitudinal changes in routinely measurable protein, lipid, energy, mineral, and oxidative variables during early pregnancy, their relationships with P_4_ and PAG, and their individual or combined prognostic and discriminatory performance. Such an approach may not only characterize the maternal biochemical phenotype associated with early pregnancy but also identify practical biomarker combinations based on widely available laboratory measurements.

Accordingly, the present study was designed to evaluate early pregnancy in ewes from three complementary perspectives. The aims were to (i) characterize temporal changes in protein, lipid, mineral, hormonal, and oxidative stress profiles during early pregnancy; (ii) determine the relationships of maternal biochemical variables with the established pregnancy indicators P_4_ and PAG, thereby defining the links between maternal systemic responses and luteal and conceptus–placental activity; and (iii) conduct an exploratory, hypothesis-generating evaluation of candidate biomarkers and biomarker combinations for their potential contribution to the early prediction or discrimination of pregnancy and assess whether their integration with P_4_ and PAG provides additional value for early pregnancy assessment. We hypothesized that early pregnancy would be associated with a temporally coordinated and measurable maternal biochemical phenotype, and that selected components of this phenotype, when evaluated together with luteal and conceptus–placental signals, could contribute to both the biological characterization and discrimination of early pregnancy.

## 2. Materials and Methods

### 2.1. Animals and Management

The presented study was conducted outside the breeding season (April–June 2025) in Antakya District of Hatay Province, located in the Southeastern Anatolia Region (36°14′53.88″ N, 36°21′11.16″ E and altitude: 67 m). The study involved 80 multiparous lactating Awassi ewes, 3–5 years old, with a mean parity of 2.86 ± 0.07, body weight (BW) of 41.21 ± 0.31 kg, and body condition score (BCS) of 2.75 ± 0.03 (with BCS ranging from 1 = thin to 5 = obese) [33] and were approximately 90 days postpartum. Throughout the experimental period, all ewes were managed under the same grazing conditions and were maintained primarily on natural pasture without concentrate supplementation. In addition to grazing, dry meadow hay and wheat straw were offered as roughage before turning out to pasture and after the ewes returned to the farm. The ewes grazed for approximately 12 h/day and were housed in a semi-open barn during the remaining period. Fresh water was available ad-libitum, and no additional mineral or vitamin supplementation was provided. The ewes were hand-milked once daily, with an average milk yield of 0.675 kg/ewe/day. Management conditions, grazing duration, milking frequency, and access to roughage and water were kept consistent throughout the experimental period. This prospective longitudinal field study followed ewes from mating through early pregnancy, with animals subsequently classified according to confirmed reproductive outcome. No experimental changes were introduced to routine feeding, housing, milking, or general husbandry. Animals remained under their normal farm management conditions and routine care and were monitored under routine farm management procedures throughout the study.

### 2.2. Estrus Synchronization and Blood Sampling

All ewes (*n* = 80) received an intravaginal sponge (Esponjavet^®^, Hipra, Amer, Spain) impregnated with 60 mg medroxyprogesterone acetate (MAP) for nine days. At sponge removal, 500 IU eCG (Oviser^®^, Hipra, Amer, Spain) and 75 μg *d*-cloprostenol sodium (PGF_2_α) (Dalmazin^®^, Fatro, Bologna, Italy) were administered intramuscularly [34]. Subsequently, fertile rams (ewe-to-ram ratio of 1:8) were kept with the ewes for 2 h twice daily from the 24th to the 72nd h after sponge removal (Figure 1). Estrus and mating activity were monitored during this period, and mated ewes were individually marked and recorded. A total of 69 ewes were confirmed to have mated, after which the rams were removed and were not reintroduced for the remainder of the study.

Approximately 8 mL of blood samples were collected from the jugular vein into vacuum gel serum tubes (Vacusera^®^, Disera, İzmir, Türkiye) to evaluate the metabolic profile and oxidative stress status in early pregnancy. Blood samples were collected from all ewes (*n* = 80) immediately before ram introduction, and this sampling point was designated as day 0 (D0). Subsequent samples were collected only from ewes with confirmed mating (*n* = 69) on days 7 (D7), 14 (D14), 21 (D21), 35 (D35), and 50 (D50) after mating (Figure 1). Samples were centrifuged at 3000× *g* for 10 min, and the separated serum was stored at −20 °C until analysis.

### 2.3. Pregnancy Diagnosis

Pregnancy was diagnosed transrectally on D35 and D50 using a real-time B-mode portable ultrasound device (Hasvet 838^®^, Hasvet, Antalya, Türkiye) equipped with a 7.5-MHz linear transducer (Figure 1). The presence of the embryo sac, embryo, heartbeat, and placentome was considered a positive pregnancy. Ewes diagnosed as pregnant on D35 but without a viable fetus on D50 were classified as having experienced pregnancy loss and were excluded from the final analysis [34]. Lambing records were used to confirm pregnancy outcome and litter size. Ewes were classified as pregnant when pregnancy was confirmed on both D35 and D50 and subsequently resulted in lambing, whereas non-pregnant ewes showed no ultrasonographic evidence of pregnancy and did not lamb.

### 2.4. Analysis of Metabolic Profile, Hormonal, and Oxidative Stress Parameters

Based on pregnancy examinations and lambing records, 32 of the 69 ewes with confirmed mating were diagnosed as pregnant and 37 as non-pregnant. Among the pregnant ewes, four experienced pregnancy loss and six carried multiple fetuses, leaving 22 ewes with confirmed singleton pregnancies. From these ewes, pregnant ewes (*n* = 22; BW = 41.95 ± 1.66 kg; BCS = 2.85 ± 0.57; Parity = 3.05 ± 0.75) carrying a single lamb and non-pregnant ewes (*n* = 22; BW = 40.48 ± 1.72 kg; BCS = 2.66 ± 0.61; Parity = 2.73 ± 0.79) were selected for the final biochemical analyses. Among the 37 confirmed non-pregnant ewes, animals with body weight and body condition score comparable to those of the pregnant group were first identified, and 22 ewes were then randomly selected from this eligible pool to improve comparability between groups (Figure 2). Field investigators responsible for mating records and pregnancy diagnosis had access to reproductive status information, whereas laboratory personnel were blinded to pregnancy-group allocation during sample analysis.

The metabolic profile included glucose, lactate dehydrogenase (LDH), C-reactive protein (CRP); protein parameters [urea, blood urea nitrogen (BUN), total protein (TP), albumin (A), globulin (G), and A/G ratio]; lipid parameters [cholesterol, triglyceride, high-density lipoprotein cholesterol (HDL-C), low-density lipoprotein cholesterol (LDL-C), LDL/HDL ratio, and very-low-density lipoprotein (VLDL)]; and mineral parameters [calcium (Ca), phosphorus (P), magnesium (Mg), and Ca/P ratio]. Serum metabolic parameters were analyzed using fully automated clinical chemistry and immunochemistry systems (Beckman Coulter, Brea, CA, USA) with manufacturer-supplied reagents, according to the methods specified in the manufacturer’s instructions. For photometric assays, measurements were performed at assay-specific wavelengths ranging from 340 to 800 nm. Detailed information on the analyzers, reagent reference/catalog numbers, analytical principles, measurement ranges, sensitivity, and precision criteria is provided in Supplementary Table S7.

Serum P_4_ concentrations were determined using a competitive-binding chemiluminescent immunoenzymatic assay (Progesterone, Beckman Coulter, Brea, CA, USA. Cat. No. 33550) on a UniCel DXI 800 Access Immunoassay System (Beckman Coulter, USA), with an analytical sensitivity of 0.10 ng/mL and a measurement range of approximately 0.10–40.0 ng/mL. The manufacturer-reported within-run and between-run coefficients of variation ranged from 6.11–11.19% to 6.59–9.57%, respectively.

Oxidative stress status was evaluated by measuring total antioxidant status (TAS; E-BC-K801-M, Elabscience, Wuhan, China) and total oxidant status (TOS; E-BC-K802-M, Elabscience, Wuhan, China) using commercial colorimetric assay kits according to the manufacturer’s instructions. TAS was determined using an ABTS-based colorimetric method at 660 nm, with a sensitivity of 0.23 mmol Trolox Equiv./L; a detection range of 0.23–2.0 mmol Trolox Equiv./L; and intra- and inter-assay coefficients of variation of 4.6% and 7.0%, respectively. TOS was determined based on the oxidation of Fe^2+^ to Fe^3+^ under acidic conditions and subsequent complex formation with xylenol orange, with absorbance measured at 590 nm. The assay had a sensitivity of 2.5 μmol H_2_O_2_ Equiv./L, a detection range of 2.5–100 μmol H_2_O_2_ Equiv./L, and intra- and inter-assay coefficients of variation of 2.3% and 3.5%, respectively. The oxidative stress index (OSI) was calculated after expressing TAS in μmol Trolox Equiv./L as follows: OSI = [TOS (μmol H_2_O_2_ Equiv./L)/TAS (μmol Trolox Equiv./L)] × 100.

Serum PAG levels were determined using a commercial ELISA kit (Alertys Ruminant Pregnancy Test, IDEXX Laboratories, Westbrook, ME, USA; Cat. No. 99-41169) according to the manufacturer’s instructions. Briefly, samples were incubated in anti-PAG antibody-coated microplates, followed by sequential incubation with detection antibody, horseradish peroxidase conjugate, and tetramethylbenzidine substrate. Absorbance was measured at 450 nm using a microplate reader (Lisascan EM, Erba Mannheim, Brno, Czech Republic). PAG levels were expressed semi-quantitatively as optical density (O.D.) values calculated using the sample-minus-negative control (S-N) method. The intra- and inter-plate coefficients of variation for the positive controls were 2.7% and 6.3%, respectively.

### 2.5. Statistical Analysis

G*Power software version 3.1 was used for the sample size calculation using a repeated-measures ANOVA framework for the group-by-time interaction. The analysis considered a small-to-medium effect size (Cohen’s f = 0.16), an alpha error probability of 0.05, a power (1 − β) of 0.80, two groups (pregnant and non-pregnant), six measurements (days 0, 7, 14, 21, 35, and 50), a correlation of 0.5 among repeated measures as an estimated baseline assumption, and a non-sphericity correction (ε) of 1.0. The calculation indicated that a minimum of 44 ewes was required. The individual ewe was considered the experimental unit.

The assumptions of normality and homogeneity of variance were assessed for the quantitative variables (metabolic profile, serum P_4_ concentration, PAG level, and oxidative stress parameters) using the Shapiro–Wilk and Levene tests, respectively. Differences in the metabolic profile, oxidative stress parameters, serum P_4_ and PAG levels between groups over time were evaluated using a linear mixed-effects model for repeated measures. The repeated-measures model was specified as follows: $Y_{ijkl}=\mu +G_{i}+D_{j}+{\left(G\;\times D\right)}_{k}+E_{l}+e_{ijkl}$ where $Y_{ijkl}$ was the dependent variable; μ was the overall mean; $G_{i}\;$was the fixed effect of group (*i* = pregnant or non-pregnant); $D_{j}$ was the fixed effect of sampling day (*j* = days 0, 7, 14, 21, 35, and 50); ${\left(G\;\times \;D\right)}_{k}$ was the group-by-day interaction; $E_{l}$ was the random effect of ewe; and $e_{ijkl}$ was the residual error. Ewe was included as a random effect, whereas group, sampling day, and the group-by-day interaction were included as fixed effects. A variance-components covariance structure was selected because it yielded the lowest Akaike Information Criterion (AIC). When significant effects were detected, Bonferroni-adjusted simple-effects comparisons were performed. Residual normality and homoscedasticity were assessed using normal quantile–quantile (Q-Q) plots and residual diagnostics; no serious violations were observed. The final analytical dataset contained no missing biochemical or hormonal observations across the six time points. Pearson correlations were calculated at days 21, 35, and 50 for the pooled cohort and the pregnant-only subgroup and were visualized as heatmaps.

Exploratory receiver operating characteristic (ROC) analyses were performed on days 0, 7, 14, and 21 to evaluate the day-specific discrimination of final pregnancy outcome by the selected variables. Findings from day 0 were interpreted as prognostic performance for subsequent maintained pregnancy outcome, whereas findings from days 7, 14 and 21 were described as pregnancy-status discrimination within the study cohort. Variables showing significant group differences during the longitudinal analysis were included in univariable ROC analyses. For each variable, the area under the curve (AUC), cohort-derived optimal cutoff, sensitivity, specificity, and corresponding 95% confidence intervals were calculated. Multivariable panels were evaluated at days 7, 14, and 21. Variables with significant univariable discrimination were prioritized according to day-specific AUCs, and parsimonious panels were constructed from biologically distinct, non-redundant variables. Selected combinations were fitted using multivariable logistic regression, and the resulting predicted probabilities were used to generate combined ROC curves and calculate AUCs. All statistical analyses were performed using Stata/SE 15.1. The scikit-learn library was used for multivariable logistic regression and ROC analyses [35], pandas for data management [36], and matplotlib for data visualization [37]. Statistical significance was set at *p* < 0.05, and 0.05 ≤ *p* ≤ 0.10 was considered a tendency.

## 3. Results

### 3.1. Temporal Changes in Maternal Metabolic, Hormonal, and Oxidative Profiles During Early Pregnancy

Pregnancy was associated with coordinated changes in several components of the maternal biochemical profile (Figure 3, Figure 4, Figure 5 and Figure 6; Supplementary Tables S1–S6). The most consistent differences involved protein metabolism. Pregnant ewes had lower TP and globulin concentrations and a higher A/G ratio from the early post-mating period onward, whereas albumin was unaffected by pregnancy status (Figure 3A–D; Supplementary Table S1). BUN and urea varied markedly over time, with the principal pregnancy-related difference emerging at D50, when both were lower in pregnant ewes (Figure 3E,F).

Lipid and mineral profiles also showed pregnancy-associated changes. Cholesterol, triglyceride, and HDL-C concentrations were higher overall in pregnant ewes, while differences in LDL-C were concentrated between D7 and D21 and VLDL remained higher at several post-mating time points (Figure 4; Supplementary Table S2). Among minerals, P was higher at several sampling points, and the Ca/P ratio was lower in pregnant ewes from D7 to D35, whereas Mg was unaffected by pregnancy status (Figure 5; Supplementary Table S3).

Among the remaining metabolic variables, LDH showed one of the clearest early pregnancy-related responses, with higher concentrations in pregnant ewes from D7 to D35 before converging at D50. Glucose did not differ between groups, whereas CRP was modestly lower overall in pregnant ewes (Figure 6A–C; Supplementary Table S4). The oxidative profile also differed according to pregnancy outcome. TOS showed a significant time-dependent group response, with lower concentrations in ewes that subsequently became pregnant at D0 and D14, whereas TAS was unaffected by pregnancy status. OSI was lower overall in pregnant ewes (Figure 6D–F; Supplementary Table S5).

The established pregnancy-associated markers showed pronounced temporal separation between groups. Serum P_4_ was affected by pregnancy status, day, and their interaction (all *p* < 0.001). Concentrations were low and comparable between groups at D0 but were consistently higher in pregnant ewes from D7 to D50, reaching their highest values around D21. At D21, serum P_4_ concentrations were below 0.5 ng/mL in all 22 ewes included in the final non-pregnant group. PAG, measured from D21 onward, also showed significant group, day, and group × day effects (all *p* < 0.001). PAG concentrations were higher in pregnant ewes at D21, D35, and D50 and increased progressively as pregnancy advanced (Figure 7; Supplementary Table S6).

### 3.2. Associations of the Maternal Biochemical Profile with P_4_ and PAG

Correlation patterns differed markedly between the pooled population and the analysis restricted to pregnant ewes (Figure 8 and Figure 9; Supplementary Dataset). In the pooled population, P_4_ and PAG were moderately correlated at D21 (r = 0.353, *p* = 0.019), with substantially stronger correlations at D35 (r = 0.816, *p* < 0.001) and D50 (r = 0.844, *p* < 0.001). At D21, P_4_ showed significant associations with several biochemical variables, including positive correlations with LDH and A/G and negative correlations with TP, globulin, and Ca, whereas PAG was associated only with OSI. By D35 and D50, both P_4_ and PAG exhibited broader and largely overlapping relationships with the maternal biochemical profile. These were characterized mainly by negative associations with TP, globulin, and Ca and positive associations with VLDL and A/G. Additional associations involved P and Ca/P at D35 and BUN and urea at D50 (Figure 8A and Figure 9A).

When the analysis was restricted to pregnant ewes, the correlation structure was markedly attenuated. P_4_ and PAG were not significantly correlated at D21, D35, or D50, and most associations with maternal biochemical variables observed in the pooled population disappeared. Only a few isolated relationships remained, including P_4_ with ICA at D21, P_4_ with P and A/G at D50, and PAG with LDL/HDL at D50 (Figure 8B and 9B). Overall, the stronger correlations observed in the pooled analysis were substantially reduced when pregnancy status was held constant.

### 3.3. Exploratory Prognostic and Discriminatory Performance of Candidate Maternal Biomarkers During the Early Post-Mating Period

Following longitudinal evaluation, variables showing clear and statistically significant differences between pregnant and non-pregnant ewes during the sampling period were selected for exploratory day-specific univariable ROC analyses to assess their potential prognostic or discriminatory value. The apparent prognostic or discriminatory performance of candidate maternal biomarkers varied markedly with sampling day (Figure 10, Figure 11, Figure 12, Figure 13 and Figure 14; Supplementary Dataset). At D0, TOS and OSI showed the highest apparent prognostic performance for subsequent maintained pregnancy outcome. TOS yielded an AUC of 0.939 (*p* < 0.001) at a cutoff of ≤21.19 µmol H_2_O_2_ Equiv./L, with 85.71% sensitivity and 100% specificity, whereas OSI yielded an AUC of 0.918 (*p* < 0.001) at a cutoff of ≤3.29, with the same sensitivity and specificity. CRP showed moderate discrimination (AUC = 0.750), whereas P showed lower overall performance (AUC = 0.695) despite 100% sensitivity at >3.1 mg/dL, which was accompanied by only 27.27% specificity (Figure 10).

By D7, P_4_ and LDH emerged as the strongest individual predictors of subsequent pregnancy status. P_4_ achieved an AUC of 0.948 (*p* < 0.001), with a cutoff of >1.57 yielding 95.45% sensitivity and 81.82% specificity. LDH showed similarly high performance among the non-hormonal markers (AUC = 0.915, *p* < 0.001), with 100% sensitivity and 68.18% specificity at >302 U/L. Ca/P and P also showed good discrimination, whereas A/G and CRP showed moderate performance and several protein and lipid variables provided weaker but significant discrimination (Figure 11).

At D14, discriminatory information extended across endocrine, protein, lipid, mineral, and oxidative domains. P_4_ (AUC = 0.857), A/G (AUC = 0.850), and cholesterol (AUC = 0.838) showed the strongest performance within the complete biochemical dataset. The selected cutoffs for P_4_ (>2.77), A/G (>0.48), and cholesterol (>46 mg/dL) yielded sensitivities and specificities of 86.36% and 72.73%, 100% and 50%, and 72.73% and 95.45%, respectively. TOS and OSI also showed high apparent discrimination (AUC = 0.918 for both), while several lipid and mineral variables provided significant but more moderate performance (Figure 12).

By D21, separation between pregnant and non-pregnant ewes became more pronounced. P_4_ provided complete discrimination in the present dataset (AUC = 1.000, *p* < 0.001), with a cutoff of >0.31 yielding 100% sensitivity and 100% specificity. A/G also showed excellent discrimination (AUC = 0.916), followed by globulin (AUC = 0.842) and TP (AUC = 0.832), whereas Ca and LDH showed more moderate performance (Figure 13).

Multivariable modelling further evaluated whether maternal biochemical variables provided complementary information when combined with established pregnancy markers. Among variables demonstrating statistically significant univariable discrimination, those with higher day-specific AUCs were prioritized for exploratory multivariable modelling. Parsimonious panels were then constructed by combining markers representing distinct biological domains while avoiding the simultaneous inclusion of closely overlapping or mathematically related variables to evaluate whether these biochemical measures provided information complementary to that obtained from P_4_ and PAG.

At D7, the LDH + A/G + Ca/P model yielded an AUC of 0.928, which increased to 1.000 after inclusion of P_4_. The integrated model achieved 95.45% sensitivity, 100% specificity, and 97.73% classification accuracy. At D14, the A/G + cholesterol + Ca/P model yielded an AUC of 0.863, increasing to 0.919 with P_4_, with 81.82% sensitivity, 86.36% specificity, and 84.09% classification accuracy. At D21, the addition of PAG increased the AUC of the A/G + LDH model from 0.835 to 0.870. The PAG + P_4_ model achieved an AUC of 1.000, although P_4_ alone had already provided complete discrimination at this time point (Figure 14; Supplementary Dataset).

## 4. Discussion

The present study demonstrates that successful early pregnancy in ewes is accompanied by a coordinated maternal biochemical and oxidative response over time. The main findings were that pregnancy was associated with changes in protein, lipid, mineral, LDH, and redox-related variables. Some differences were already present around mating, whereas others appeared after pregnancy establishment. Maternal biochemical variables showed stronger associations with P_4_ and PAG when pregnant and non-pregnant ewes were analyzed together. Most of these associations became weaker when only pregnant ewes were evaluated. This suggests that maternal biochemical status, P_4_, and PAG represent related but distinct aspects of early pregnancy physiology. Finally, exploratory analyses identified several routinely measurable maternal variables with promising apparent prognostic and discriminatory performance during the early post-mating period. Selected combinations also suggested potential complementary value when evaluated together with established pregnancy markers. These results should be regarded as hypothesis-generating candidate-biomarker findings rather than validated diagnostic evidence. Collectively, these findings suggest that maternal metabolic status, luteal activity, and conceptus–placental function provide complementary information on early pregnancy establishment.

### 4.1. Maternal Metabolic and Oxidative Adaptations Associated with Early Pregnancy

Pregnancy was associated with one of the most pronounced changes in protein metabolism. Pregnant ewes had lower TP and globulin concentrations from the early post-mating period onward and a consistently higher A/G ratio, whereas albumin remained largely unaffected by pregnancy status (Figure 3A–D). This pattern indicates that the reduction in TP was primarily related to the globulin fraction rather than to generalized hypoproteinemia. Oztabak et al. [38] found lower TP and globulin concentrations during the final four weeks of gestation in pregnant ewes compared with non-pregnant controls. Zywicki et al. [39] similarly observed lower TP concentrations during late gestation than during the follicular phase in non-pregnant ewes, while Batavani et al. [40] described a progressive decline in serum proteins as gestation advanced. Direct information on the A/G ratio is more limited, although Fitriani et al. [41] observed numerically lower globulin and higher A/G values in pregnant ewes. Most of these studies focused on late gestation or comparisons among different physiological states. The emergence of a similar pattern as early as day 7 in the present study therefore suggests that changes in circulating protein composition may begin considerably earlier in pregnancy. This interpretation is consistent with the serum proteomic findings of Zhai et al. [26], who demonstrated a clear separation between pregnant and non-pregnant ewes on day 21 and identified multiple pregnancy-related proteins with differential abundance. The lower globulin and higher A/G ratio observed here may therefore represent routine biochemical manifestations of a broader reorganization of the circulating protein pool. Nevertheless, globulin is a heterogeneous fraction comprising immunoglobulins, transport proteins, acute phase proteins, and numerous other circulating proteins [42]. These changes should therefore not be interpreted as direct evidence of systemic immunosuppression or maternal immune tolerance without characterization of specific protein fractions or immunological markers.

Changes in nitrogen metabolism became more evident later in the observation period. The clearest differences in BUN and urea occurred at D50, when both were lower in pregnant ewes (Figure 3E,F). This finding closely agrees with Goldansaz et al. [25], who reported lower serum urea concentrations in pregnant ewes on day 50 and identified urea as a component of a metabolite panel associated with pregnancy. Lower circulating urea during gestation may reflect more than differences in protein intake. Benlamlih and De Pomyers [43] demonstrated in sheep that pregnancy reduced renal urea excretion while increasing fractional endogenous urea recycling. This response is consistent with the capacity of ruminants to conserve nitrogen through gastrointestinal urea recycling and subsequent reutilization rather than urinary loss [44,45]. Thus, the lower BUN and urea concentrations observed at D50 may reflect a shift in maternal nitrogen metabolism toward greater conservation and reutilization as pregnancy progresses. However, the present data cannot distinguish the relative contributions of hepatic urea production, renal handling, and gastrointestinal urea recycling.

Pregnant ewes also showed a distinct lipid profile, with generally higher cholesterol, triglyceride, and HDL-C concentrations, higher LDL-C mainly from D7 to D21, and a marked temporal response in VLDL (Figure 4). Mazur et al. [46] demonstrated substantially higher plasma triglyceride and VLDL triglyceride concentrations in adequately fed pregnant ewes, together with an increased triglyceride secretion rate. The attenuation of these responses by energy restriction in the same study also emphasized the strong influence of nutritional and energy status on lipid metabolism during pregnancy. Nazifi et al. [47] reported higher cholesterol, triglyceride, HDL-C, and VLDL concentrations shortly before parturition, which is directionally consistent with several of the lipid changes observed here. However, Zywicki et al. [39] found lower cholesterol concentrations during late gestation, indicating that circulating lipid responses are not uniform throughout pregnancy and may vary with gestational stage, breed, nutritional status, lactation, and energy balance. These considerations are particularly relevant to the present study because the ewes were lactating and maintained under pasture-based conditions during the nonbreeding season. The early changes observed in circulating lipids are also compatible with extensive lipid remodelling within the uterus and conceptus. O’Neil and Spencer [11] demonstrated substantial changes in endometrial and uterine luminal lipid composition on day 14, while de Brun et al. [21] identified conceptus-associated alterations in uterine pathways related to energy metabolism and fatty acid oxidation. Taken together, the present serum findings may represent a systemic component of broader changes in maternal lipid transport and utilization during early pregnancy. They do not, however, demonstrate direct transfer of circulating lipids to the uterus or increased substrate availability for luteal steroidogenesis.

Mineral metabolism showed an equally distinct pattern. Pregnant ewes had lower Ca, higher P at several sampling points, and consequently a lower Ca/P ratio from D7 to D35, whereas Mg was unaffected by pregnancy status (Figure 5). These findings are biologically plausible because Ca and phosphate availability are actively regulated within the maternal conceptus environment. Stenhouse et al. [14] demonstrated that phosphate abundance and phosphate regulatory pathways change across ovine gestation and that relevant transport systems are expressed in the endometrium, conceptus, and placenta. The same group subsequently demonstrated dynamic regulation of Ca-binding proteins, Ca transporters, and vitamin D-related pathways in ovine reproductive tissues [48]. Experimental evidence further indicates that P_4_ and interferon tau (IFN-τ) contribute to the regulation of these mineral-related pathways during the period surrounding implantation [13]. Thus, the higher circulating P and lower Ca/P ratio observed in pregnant ewes may be compatible with altered mineral handling during early conceptus development. Circulating Ca and P, however, are also influenced by dietary intake, gastrointestinal absorption, renal handling, bone metabolism, and endocrine regulation [49]. Previous systemic studies have not shown a uniform response. Zywicki et al. [39] reported lower P concentrations during late gestation, while Yildiz et al. [50] observed reductions in both Ca and inorganic P, particularly in ewes carrying twins. This discrepancy may partly reflect gestational stage because the present study focused on conceptus elongation, implantation, and early placental development rather than the later period of intensive fetal mineral accretion. Importantly, P was already higher at D0 in ewes that subsequently became pregnant (Figure 5B). The early P difference therefore cannot be attributed solely to established pregnancy or conceptus signaling. Instead, higher peri-mating P status, or a broader nutritional and metabolic phenotype associated with it, may have characterized ewes with greater reproductive competence. This possibility is indirectly supported by Senosy et al. [51], who reported beneficial reproductive responses to phosphorus supplementation in anestrous ewes. Nevertheless, the present findings do not establish a causal role for P. The persistence of higher P together with a lower Ca/P ratio after mating may instead reflect the combined contribution of a pre-existing maternal mineral phenotype and subsequent adaptation associated with pregnancy.

Peripheral glucose concentrations did not differ between pregnant and non-pregnant ewes (Figure 6A), but this does not indicate that carbohydrate metabolism remained unchanged. Maternal circulating glucose is subject to tight homeostatic regulation, while nutrient availability and glucose transport within the uterus can change substantially during early pregnancy. Satterfield et al. [7] demonstrated that progesterone increased uterine luminal glucose availability and the expression of glucose transporters in ewes. The ovine conceptus also actively metabolizes glucose and fructose, and recent work has shown that a substantial proportion of these hexoses is converted to lactate [5]. Moses et al. [6] further demonstrated increasing lactate abundance in the uterine lumen during the preimplantation period and expression of both LDHA and LDHB by the conceptus. Against this metabolic background, LDH was one of the most pronounced early biochemical responses in the present study. LDH concentrations were similar between groups at D0 but became higher in pregnant ewes from D7 to D35 (Figure 6B). Although this temporal pattern is biologically compatible with the increasing importance of lactate metabolism during conceptus development, circulating LDH cannot be assumed to originate from the conceptus. LDH is widely distributed among tissues, and total serum LDH has limited tissue specificity because multiple tissues contribute to circulating activity [52]. The observed increase should therefore be considered a systemic biochemical correlate associated with successful pregnancy establishment rather than evidence of direct release of conceptus-derived LDH into the circulation. Importantly, unlike P and TOS, LDH showed no pre-existing difference at D0 and diverged only after mating. This temporal feature strengthens its potential relevance as a dynamic maternal biomarker associated with early pregnancy.

The oxidative profile provided a complementary perspective because differences were evident both before and after pregnancy establishment. TOS varied over time between outcome groups, OSI was generally lower in pregnant ewes, and TAS remained largely unchanged (Figure 6D–F). Notably, TOS was already lower at D0 in ewes that subsequently maintained pregnancy. This difference cannot therefore be considered a response induced by the conceptus and may instead reflect a pre-existing maternal redox phenotype associated with subsequent reproductive success. Samaei et al. [53] similarly found higher malondialdehyde (MDA) and nitric oxide (NO) concentrations at mating in ewes that subsequently failed to become pregnant than in those that later lambed. This relationship is biologically consistent with the importance of redox balance for luteal function and pregnancy maintenance [54]. Al-Gubory et al. [55] also demonstrated dynamic regulation of antioxidant enzyme systems within the ovine CL during pregnancy. In contrast, oxidative burden may increase as gestation advances. Garrel et al. [56] demonstrated increasing oxidative damage in ovine placentomes, while El-Sayed et al. [57] reported increased serum MDA during late pregnancy. These observations highlight the need to distinguish the pre-existing D0 redox difference from adaptations that emerge after pregnancy establishment. Finally, the absence of a significant TAS response in the present study suggests that the lower OSI was more likely associated with a lower oxidant burden than with an increase in systemic antioxidant capacity.

Serum P_4_ concentrations in pregnant ewes increased progressively from D7 to D21 and then gradually declined toward D50, although they remained significantly higher than those in non-pregnant ewes at all post-mating sampling points (Figure 7A). This temporal pattern was consistent with previous findings in pregnant ewes [34,58]. The decline after D21 may reflect physiological variation in luteal steroidogenic activity and peripheral P_4_ clearance rather than luteal insufficiency, particularly because pregnancy viability was maintained through D50. Nevertheless, as only one blood sample was collected from each ewe at each stage, a contribution from the episodic nature of ovine P_4_ secretion cannot be excluded [59]. In contrast, PAG optical density values increased progressively from D21 to D50, consistent with advancing fetoplacental development (Figure 7B). This pattern agrees with previous studies reporting a progressive increase in circulating PAG levels with advancing gestation in different sheep breeds [27,29,30,60].

### 4.2. Relationships of the Maternal Biochemical Phenotype with P_4_ and PAG

Relationship analyses provided additional insight into how the maternal biochemical phenotype is linked to established pregnancy-associated signals. When all animals were analyzed together, the correlation between P_4_ and PAG became progressively stronger from D21 to D50, and both markers showed increasingly similar associations with several biochemical variables over time (Figure 8 and Figure 9). This temporal pattern has a plausible biological context but should be interpreted cautiously because the magnitude of the pooled correlations may largely reflect separation between pregnancy groups. P_4_ primarily reflects sustained luteal steroidogenic activity [28], whereas PAGs originate from trophoblast-derived placental cells and become increasingly detectable in maternal circulation as placental development progresses [61]. Serum PAG concentrations are known to increase throughout early pregnancy in ewes, consistent with the progressive rise observed in the present study [29,30]. A positive relationship between circulating P_4_ and PAG has also been reported during pregnancy [60], although this association is not consistent across studies, as shown by the absence of a significant relationship reported by Ranilla et al. [62]. These differences may reflect gestational stage, assay-specific PAG populations, and physiological variation among animals.

At D21, in the pooled analysis, P_4_ showed broader associations with the maternal biochemical profile than PAG, particularly with LDH, A/G, TP, globulin, and Ca (Figure 9A). This pattern may be biologically compatible with the earlier establishment of the luteal endocrine environment and its influence on uterine function and nutrient availability. Experimental studies in ewes have shown that increased P_4_ exposure during early pregnancy enhances uterine glucose and amino acid availability and alters the expression of nutrient transporters and factors involved in conceptus development [7,9]. Thus, the associations observed at D21 may reflect both pregnancy group separation and coordinated changes in luteal function, nutrient partitioning, and maternal metabolism during pregnancy establishment. They should not, however, be interpreted as evidence that P_4_ directly determines circulating TP, globulin, LDH, or mineral concentrations.

By D35 and D50, in the pooled analysis, both P_4_ and PAG showed broader and increasingly overlapping associations with protein, lipid, and mineral variables. This coincided with the progressive rise in circulating PAG and with greater metabolic separation between pregnant and non-pregnant ewes. The associations of PAG with VLDL, A/G, TP, globulin, Ca, BUN, and urea are therefore more appropriately interpreted as parallel features of advancing pregnancy rather than as direct metabolic effects of PAG (Figure 8A). As pregnancy progresses, maternal nutrient allocation and nitrogen, lipid, and mineral metabolism are reorganized to support conceptus and placental development [63,64]. Increasing PAG concentrations may therefore track this physiological transition without directly driving the metabolic changes.

A key interpretive finding came from the analysis restricted to pregnant ewes, which was used as a stratified complement to the pooled analysis. Under this condition, no significant correlation between P_4_ and PAG was observed at D21, D35, or D50, and most associations with maternal biochemical variables were markedly attenuated (Figure 8B and Figure 9B). This suggests that many of the strong correlations observed in the pooled analysis were driven largely by the shared separation between pregnant and non-pregnant ewes across several physiological domains. Once this between-group variation was removed, the remaining relationships were much weaker, suggesting that, within the limits of the subgroup sample size, the present data do not support tight individual-level coupling among luteal activity, trophoblast-associated PAG secretion, and maternal metabolic adaptation within established pregnancies. Evidence in cattle that greater P_4_ exposure can accelerate conceptus development and advance the appearance of PAG in maternal circulation supports a biological link between these systems without implying a direct one-to-one relationship [65].

Maternal biochemical variables therefore may represent an additional and partly independent component of the pregnancy phenotype. Their weak correlations with P_4_ or PAG within pregnant ewes do not indicate limited biological relevance but rather suggest that they capture systemic adaptations that are not fully represented by endocrine or placental markers alone. This distinction is important for biomarker development. The different temporal characteristics of P_4_ and PAG in early pregnancy assessment [27], together with successful pregnancy classification using multi-metabolite serum panels [25,26], provide a rationale for exploring combinations of biologically non-redundant information. Accordingly, P_4_, PAG, and selected maternal biochemical indices may provide complementary information on early pregnancy, although their incremental discriminatory value should be confirmed through direct comparison with single-marker models and appropriate internal or external validation. Given the number of correlation tests performed, isolated associations should be interpreted as exploratory and require confirmation in independent populations.

### 4.3. Exploratory Prognostic and Discriminatory Value of Maternal Biochemical Variables During Early Pregnancy

The exploratory individual and multivariable ROC analyses collectively suggested an apparent time-dependent transition from prediction of subsequent pregnancy establishment to discrimination of an already established pregnancy within the studied cohort. This distinction is important for interpreting the earliest sampling points. Before maternal recognition of pregnancy, a variable associated with the eventual pregnancy outcome cannot necessarily be regarded as a pregnancy-specific marker; instead, it may reflect a maternal physiological state that favors or accompanies successful establishment of pregnancy. Accordingly, within the present dataset, the D0 findings are more appropriately interpreted as hypothesis-generating prognostic signals rather than validated predictors, whereas the D7, D14 and D21 profiles may provide progressively greater information for pregnancy discrimination.

At D0, TOS and OSI showed the strongest apparent prognostic performance among the evaluated variables (Figure 10). Because sampling preceded ram introduction, however, these differences cannot have originated from pregnancy itself. Rather, the direction of the identified cut-offs suggests that a lower peri-mating oxidant burden was associated with a greater probability of subsequently establishing pregnancy. This interpretation is supported by recent observations in Gray Shirazi ewes, in which animals that subsequently remained non-pregnant had higher MDA and NO concentrations at mating than ewes that later maintained singleton or twin pregnancies [53]. Nevertheless, the D0 TOS and OSI findings should be interpreted cautiously for two reasons. First, OSI is an index derived from TOS and TAS that reflects the balance between oxidant and antioxidant systems; therefore, TOS and OSI should not be regarded as completely independent biological signals. Second, D0 sampling was performed after completion of the synchronization protocol and therefore does not represent an untreated physiological baseline. Progestagen exposure, gonadotropic stimulation, luteal regression, follicular development, and the transition to synchronized estrus may themselves influence endocrine, oxidative, and metabolic status [66,67]. Although all ewes were subjected to the same synchronization protocol, individual variation in the physiological response to synchronization may have contributed to the differences observed at D0. Consequently, the D0 redox profile may reflect a combination of pre-existing maternal physiology, responsiveness to reproductive management, and peri-mating reproductive status. Therefore, the D0 redox differences are more appropriately interpreted not as early biomarkers arising from pregnancy itself, but as candidate predictors reflecting a peri-mating redox phenotype that may be associated with subsequent reproductive success and may also be influenced by synchronization and the accompanying reproductive transition.

By D7, P_4_ and LDH showed the highest apparent individual discriminatory performance within the present dataset (Figure 11). The apparently high performance of P_4_ at this stage is biologically plausible but should not be equated with direct pregnancy diagnosis. At D7, luteal activity is present in both ewes that will ultimately maintain pregnancy and those that may subsequently undergo luteolysis or embryonic loss. Therefore, P_4_ at this stage more likely reflects the quality of the early luteal environment and its suitability for conceptus development. Experimental studies in ewes have demonstrated that increased early post-mating P_4_ exposure advances conceptus growth and elongation and alters the endometrial environment required for pregnancy establishment [9,68]. The apparent discriminatory performance of LDH is noteworthy because it represents a non-endocrine physiological domain. Its emergence after mating, despite the absence of a comparable discriminatory pattern at D0, may be consistent with the development of a systemic metabolic response associated with successful pregnancy establishment. However, because circulating LDH is influenced by numerous metabolic and tissue-related processes, the present findings do not support pregnancy specificity, and LDH should therefore be regarded as a candidate complementary predictor rather than an independent pregnancy-specific marker. The D7 multivariable results provide additional exploratory support for this interpretation but require cautious evaluation. The biochemical combination of LDH + A/G + Ca/P achieved an AUC of 0.928, but this remained slightly below the performance of P_4_ alone in the individual ROC analysis. Addition of P_4_ increased the model AUC to 1.000, with 21 of 22 pregnant and all 22 non-pregnant ewes correctly classified at the selected threshold (Figure 14). However, because this model was developed and evaluated within the same small, selected dataset without internal resampling or external validation, the complete separation is particularly susceptible to overfitting and optimism and should not be interpreted as validated performance. Thus, the principal finding at D7 is not that routine biochemical variables outperform the luteal signal, but rather that they may contain complementary information that could contribute to early prediction when combined with P_4_. This distinction is important because it places the biochemical profile within a biologically integrated, hypothesis-generating prediction framework rather than positioning individual metabolites as substitutes for established endocrine indicators.

By D14, the apparent discriminatory profile broadened considerably, involving protein, lipid, mineral, endocrine, and redox variables (Figure 12). This timing is biologically relevant because it coincides with rapid conceptus elongation and the period surrounding maternal recognition of pregnancy. Experimental work in sheep has demonstrated that the presence of the conceptus by D14 alters uterine transcriptional programs associated with metabolism and nutrient transport, while lipidomic and metabolomic analyses have identified substantial differences between cyclic and pregnant uterine environments at the same stage [11,21]. Against this background, the relatively high apparent discrimination observed for A/G and cholesterol may be consistent with the emergence of a broader maternal metabolic phenotype involving protein and lipid metabolism. It should not, however, be interpreted as evidence that either variable is mechanistically specific to pregnancy. Similarly, the strong apparent performance of TOS and OSI at D14 is intriguing but should be interpreted in light of their already evident differences at D0. Their D14 discrimination may therefore represent persistence or evolution of a pre-existing redox phenotype in addition to any pregnancy-induced oxidative adaptation. The multivariable analysis at D14 suggested potential complementarity across physiological domains. Combining A/G + cholesterol + Ca/P yielded an AUC of 0.863, which increased to 0.919 after incorporation of P_4_ (Figure 14). Importantly, however, sensitivity and specificity at the selected classification threshold remained approximately 82% and 86%, respectively. Thus, the higher AUC after adding P_4_ is consistent with improved apparent discrimination across the full range of possible thresholds and does not necessarily imply a corresponding improvement at the operating threshold selected here. This distinction argues against interpreting modest numerical increases in AUC as proof that a more complex panel will necessarily provide greater clinical utility. Rather, D14 may represent the interval in which endocrine and maternal metabolic information become increasingly complementary, while no single domain yet provides the complete separation observed later.

By D21, the biological and statistical pattern changed substantially (Figure 13). P_4_ provided complete in-sample separation between pregnant and non-pregnant ewes in the present population, whereas A/G showed the highest apparent discrimination among the non-hormonal variables, followed by globulin and TP. The high apparent discrimination of P_4_ at approximately one estrous cycle after mating is consistent with previous work. Karen et al. [27], for example, reported 100% sensitivity and 95.4% specificity for plasma P_4_ on day 18, while Boscos et al. [69] reported an overall diagnostic accuracy of approximately 91% using serum P_4_ in sheep. These findings also illustrate why the AUC of 1.000 observed in the present study should be considered cohort-specific rather than interpreted as generalizable discriminatory performance. Variation in luteal lifespan, timing of ovulation, persistent CL function, embryonic mortality, assay characteristics, and management conditions can all reduce separation between pregnant and non-pregnant animals under field conditions [27,69]. The apparent discrimination of protein-related variables at D21 is also biologically plausible. Zhai et al. [26] identified 29 differential serum metabolites and 14 differential proteins between pregnant and non-pregnant ewes on day 21, with subsequent analyses identifying serum amyloid-A (SAA) and afamin as potential early pregnancy-associated proteins. Although these findings do not directly validate A/G, globulin, or TP as pregnancy markers, they provide independent evidence that a detectable systemic serum protein signature has emerged by approximately D21. The present results may therefore be viewed as complementary to proteomic observations by showing that part of this systemic response may also be captured by inexpensive routine biochemical indices. The D21 multivariable models offer an exploratory counterpoint to the assumption that adding biomarkers necessarily improves discrimination (Figure 14). Addition of PAG increased the AUC of the A/G + LDH model only from 0.835 to 0.870, whereas A/G alone had already achieved higher discrimination than either of these combinations. Conversely, PAG + P_4_ achieved an AUC of 1.000 with perfect classification in the present dataset. Detection of PAG at this stage is biologically expected: El Amiri et al. [70] reported that circulating PAG was detectable in all pregnant ewes from day 20 onward with the evaluated RIA systems. However, because P_4_ alone had already achieved an AUC of 1.000 at D21, the PAG + P_4_ combination provided no measurable incremental discrimination in this cohort. Thus, the greatest apparent advantage of multi-marker integration was observed during D7-D14, when pregnancy-specific signals were still emerging; by D21, the luteal signal accounted for nearly all observable discrimination in this cohort.

Overall, these findings support the existence of a dynamic maternal biochemical signature associated with successful pregnancy establishment, but several methodological limitations should be considered, and the results should be regarded as candidate-biomarker discovery rather than clinical validation. First, the final analytical cohort was constructed after reproductive outcomes were known by excluding ewes with pregnancy loss or multiple gestations and selecting non-pregnant ewes with BW, BCS, and parity comparable to those of the singleton-pregnant group. Although this approach was intended to reduce physiological heterogeneity and facilitate characterization of the maternal biochemical phenotype associated with uncomplicated singleton pregnancy, it resulted in the evaluation of a selected, matched subpopulation rather than an unselected prospective field cohort. This selection may have increased the apparent separation between groups and limited the generalizability of the findings. In addition, pregnancy classification based on D35 and D50 ultrasonography could not distinguish failure of conception from embryonic loss occurring before the first pregnancy diagnosis, limiting the biological interpretation of the D0-D14 differences. Second, the a priori sample size calculation was performed for the repeated-measures analysis of longitudinal changes and was not intended to provide definitive statistical power for predictive biomarker validation. Furthermore, because the ROC and correlation analyses were exploratory and hypothesis-generating, formal multiplicity corrections were not applied across the extensive univariable thresholds and correlation matrices; therefore, the possibility of Type I error should be considered when interpreting nominally significant findings. The perfect or near-perfect performance of some models is particularly vulnerable to optimism and overfitting because predictor selection, model development, threshold determination, and performance estimation were conducted within the same small dataset without internal cross-validation or external testing. Apparent performance estimated from development data is generally higher than performance obtained in new animals, particularly when sample size is limited relative to model complexity [71]. This issue is illustrated well in sheep by Goldansaz et al. [25], who developed serum metabolite panels and subsequently evaluated them in independent commercial flocks; externally evaluated AUC-ROC values ranged from 0.81 to 0.93 rather than approaching perfect discrimination. Consequently, the present panels and cut-offs should not be interpreted as field-ready diagnostic algorithms but are promising primarily as exploratory, hypothesis-generating candidate signatures and a basis for further validation. Internal resampling and, more importantly, external evaluation in larger, independent, and unselected prospective cohorts encompassing different flocks, breeds, seasons, and reproductive-management conditions will be necessary before fixed cut-offs or multivariable combinations can be recommended for routine early pregnancy assessment.

## 5. Conclusions

Within the selected analytical cohort, successful early pregnancy in ewes was associated with coordinated changes in protein, lipid, mineral, LDH, and redox profiles. The temporal pattern of these changes suggests that some variables measured around mating may carry prognostic information for subsequent pregnancy establishment, whereas changes occurring after mating increasingly reflect the developing pregnancy. The attenuation of associations with P_4_ and PAG within pregnant ewes suggests that pooled correlations were partly influenced by separation between pregnancy groups and that maternal metabolic adaptation, luteal activity, and conceptus and placental activity represent related but distinct aspects of early pregnancy physiology. Exploratory analyses further suggest that routine biochemical variables, particularly when integrated with established reproductive markers, may warrant continued investigation as candidate indicators for early pregnancy assessment. However, because the present cutoff values and multivariable models were developed and evaluated within the same small, selected dataset, they should be considered exploratory findings intended to generate hypotheses and require validation in larger, independent, and unselected prospective populations across different flocks, breeds, seasons, and management conditions before clinical application.

## Figures and Tables

**Figure 1:**
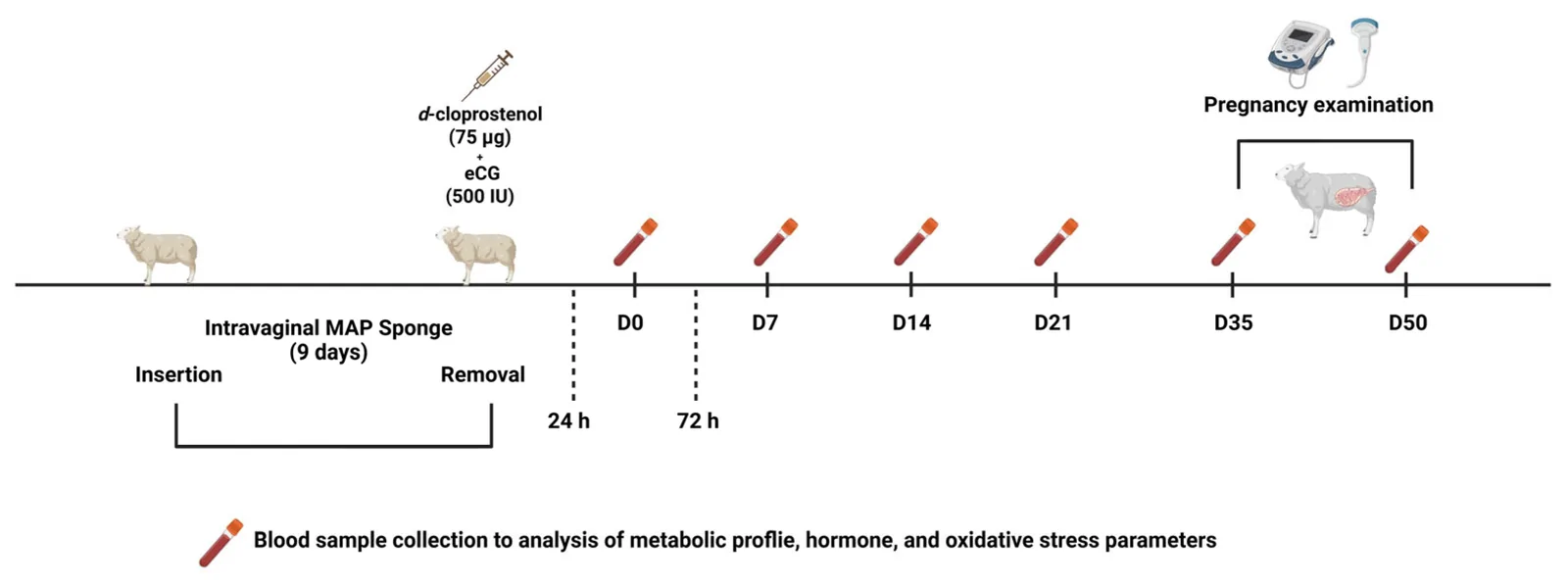
Schematic representation of the experimental design, synchronization protocol, blood sampling schedule, and pregnancy diagnosis. Ewes received an intravaginal medroxyprogesterone acetate (MAP) sponge for 9 days. At sponge removal, 75 μg d-cloprostenol and 500 IU equine chorionic gonadotropin (eCG) were administered, and mating was allowed between 24 and 72 h after sponge removal. Blood samples were collected on days (D) 0, 7, 14, 21, 35, and 50 for the assessment of metabolic, hormonal, and oxidative stress variables. Pregnancy diagnosis was performed by transrectal ultrasonography on D35 and 50.

**Figure 2:**
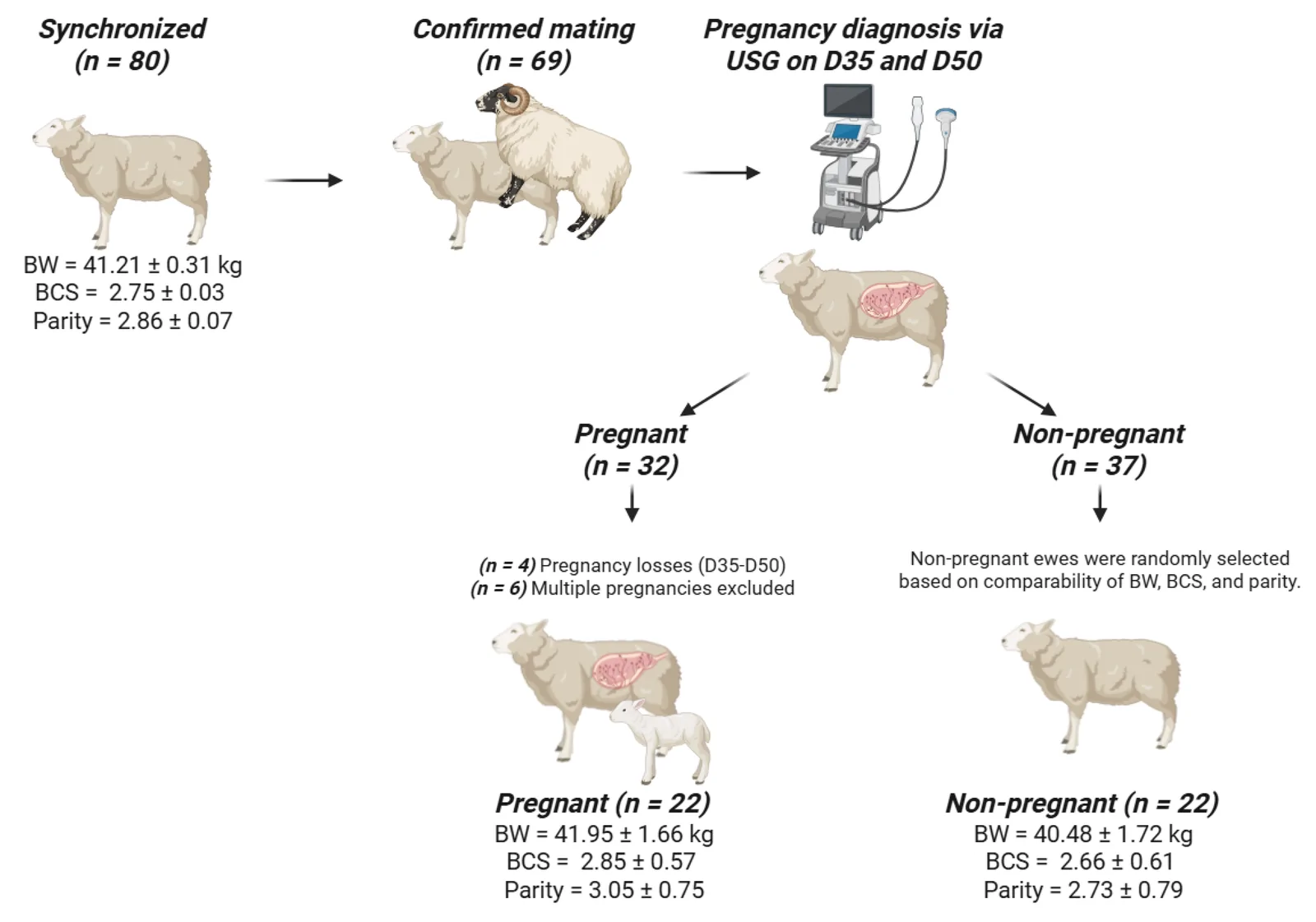
Flow diagram of ewe enrollment, pregnancy assessment, exclusions, and selection of the final singleton-pregnant and non-pregnant groups.

**Figure 3:**
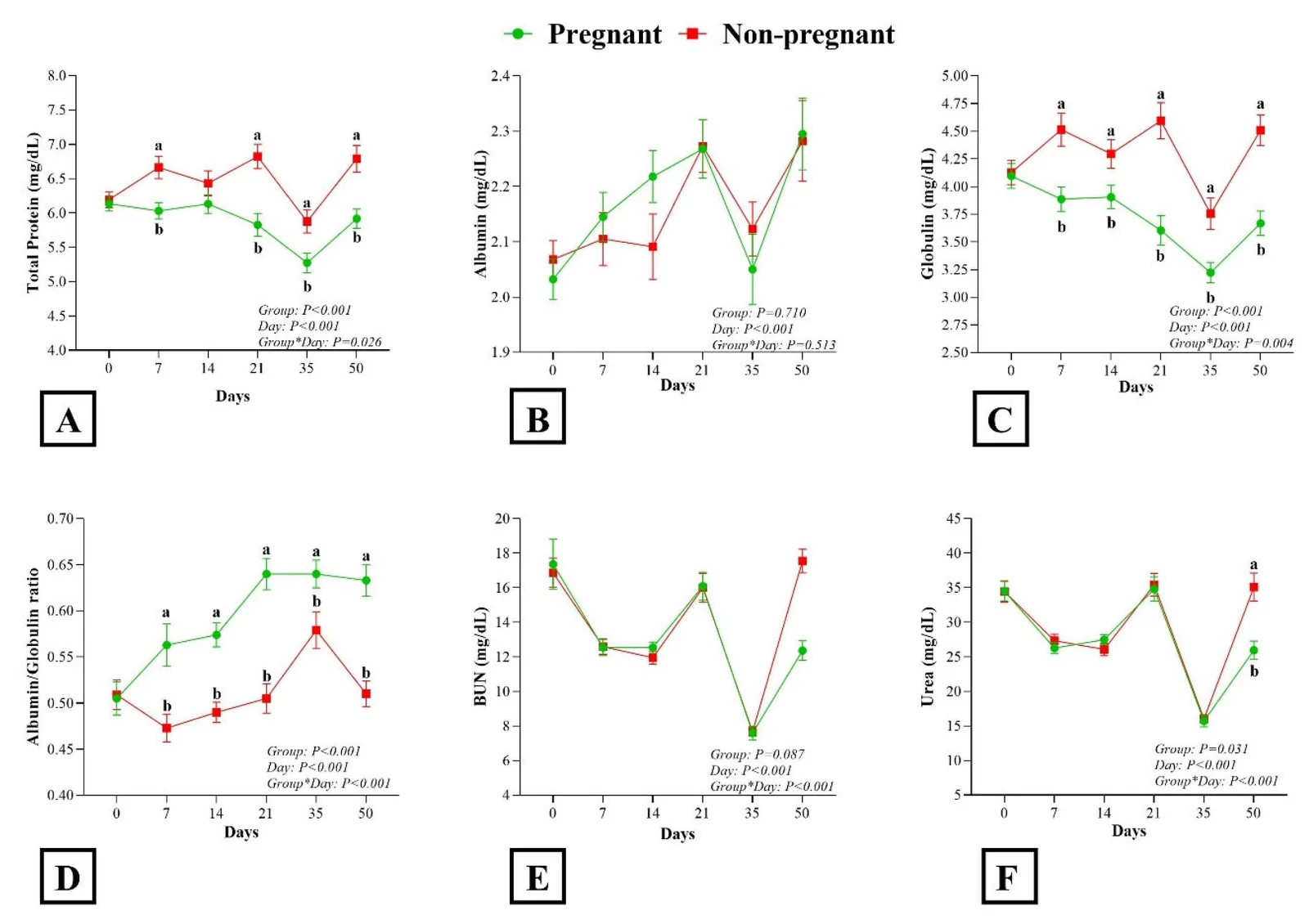
Temporal changes in protein metabolism-related variables in pregnant and non-pregnant ewes from D0 to D50. (**A**) Total protein, (**B**) albumin, (**C**) globulin, (**D**) albumin-to-globulin ratio, (**E**) blood urea nitrogen (BUN), and (**F**) urea. Data are presented as mean ± SEM. *p*-values shown in each panel indicate the effects of group, day, and group × day interaction. Different lowercase letters (a, b) indicate significant differences between pregnant and non-pregnant ewes within the same day (*p* < 0.05).

**Figure 4:**
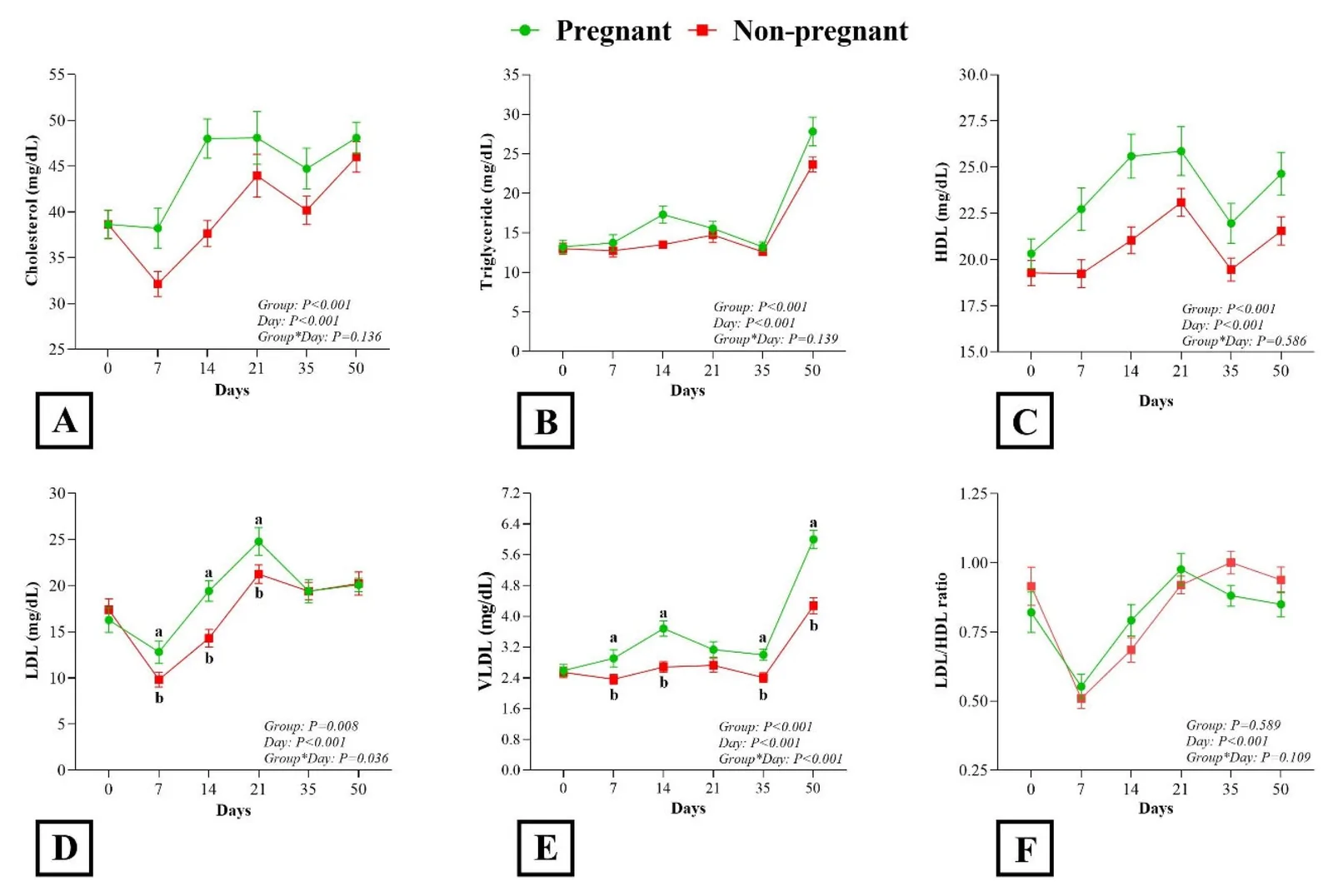
Temporal changes in lipid metabolism-related variables in pregnant and non-pregnant ewes from D0 to D50. (**A**) Cholesterol; (**B**) triglyceride; (**C**) high-density lipoprotein cholesterol (HDL-C); (**D**) low-density lipoprotein cholesterol (LDL-C); (**E**) very-low-density lipoprotein cholesterol (VLDL); and (**F**) LDL-to-HDL ratio. Data are presented as mean ± SEM. *p*-values shown in each panel indicate the effects of group, day, and group × day interaction. Different lowercase letters (a, b) indicate significant differences between pregnant and non-pregnant ewes within the same day (*p* < 0.05).

**Figure 5:**
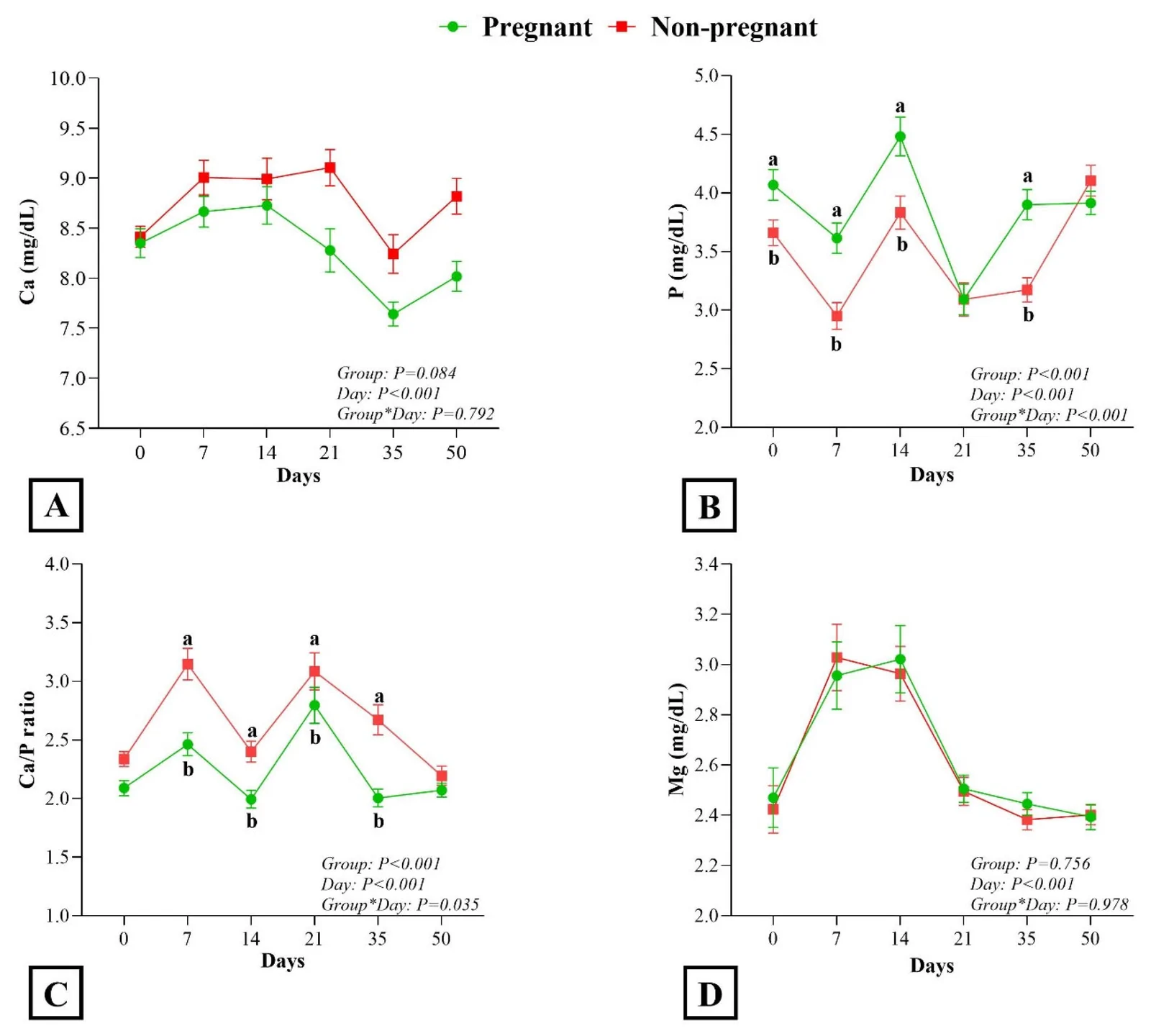
Temporal changes in mineral metabolism-related variables in pregnant and non-pregnant ewes from D0 to D50. (**A**) Calcium (Ca), (**B**) phosphorus (P), (**C**) calcium-to-phosphorus ratio (Ca/P), and (**D**) magnesium (Mg). Data are presented as mean ± SEM. *p*-values shown in each panel indicate the effects of group, day, and group × day interaction. Different lowercase letters (a, b) indicate significant differences between pregnant and non-pregnant ewes within the same day (*p* < 0.05).

**Figure 6:**
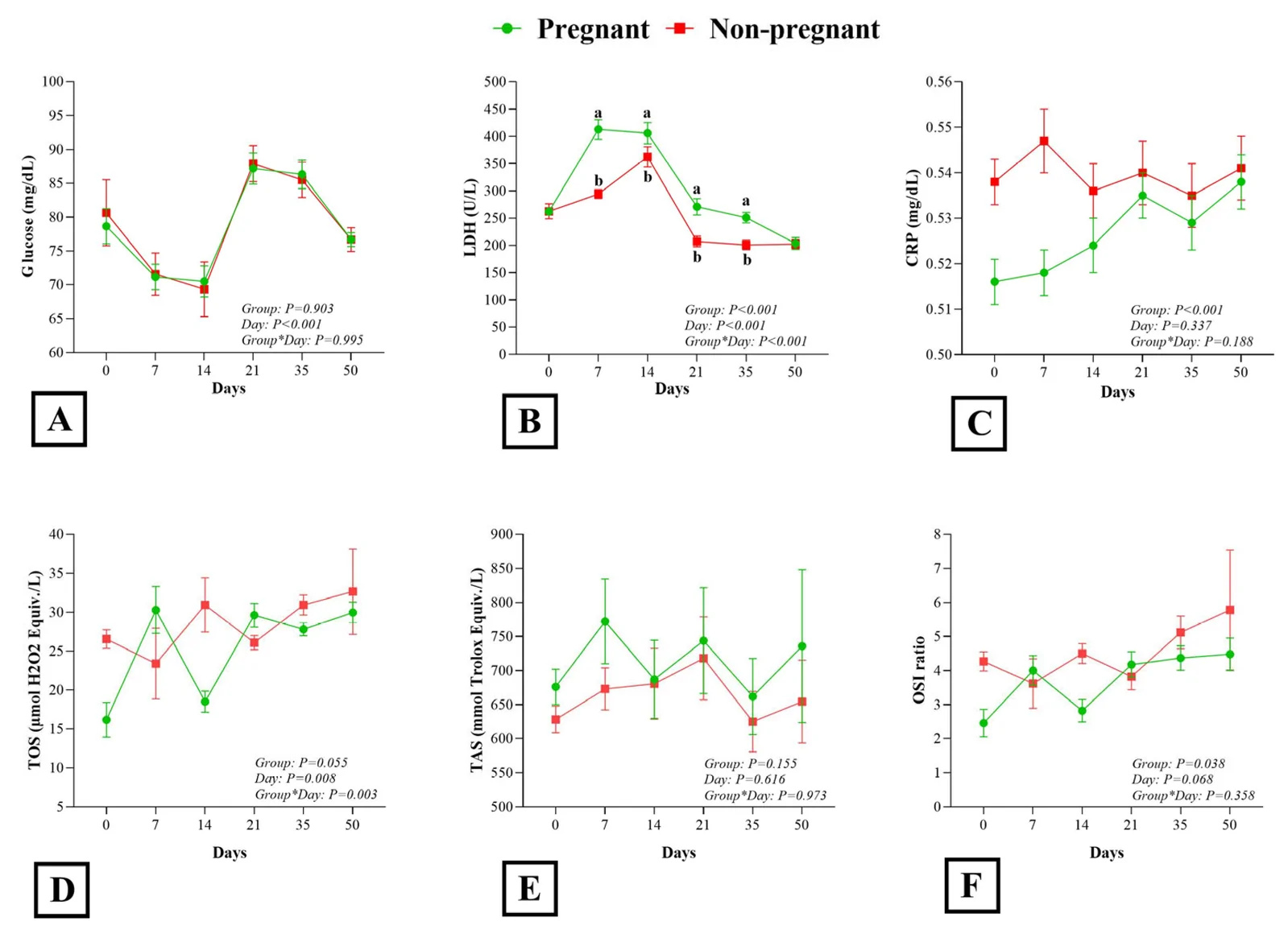
Temporal changes in selected maternal biochemical, metabolic, inflammatory, and oxidative stress variables in pregnant and non-pregnant ewes from D0 to D50. (**A**) Glucose (GLU); (**B**) lactate dehydrogenase (LDH); (**C**) C-reactive protein (CRP); (**D**) total oxidant status (TOS); (**E**) total antioxidant status (TAS); and (**F**) oxidative stress index (OSI). Data are presented as mean ± SEM. *p*-values shown in each panel indicate the effects of group, day, and group × day interaction. Different lowercase letters (a, b) indicate significant differences between pregnant and non-pregnant ewes within the same day (*p* < 0.05).

**Figure 7:**
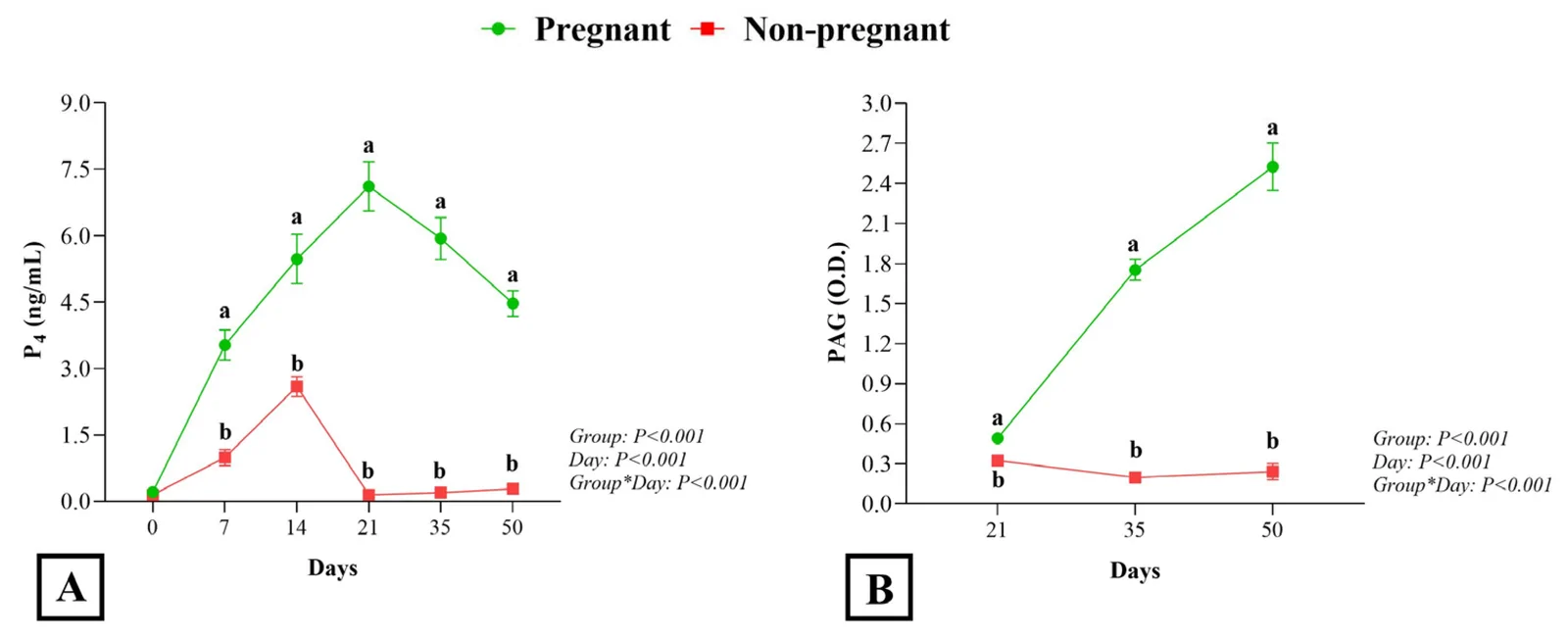
Temporal changes in maternal progesterone (P_4_) concentrations (ng/mL) and pregnancy-associated glycoprotein (PAG) optical density (O.D.) values in pregnant and non-pregnant ewes. (**A**) Serum P_4_ concentrations from D0 to D50 and (**B**) PAG values from D21 to D50. Data are presented as mean ± SEM. *p*-values shown in each panel indicate the effects of group, day, and group × day interaction. Different lowercase letters (a, b) indicate significant differences between pregnant and non-pregnant ewes within the same day (*p* < 0.05).

**Figure 8:**
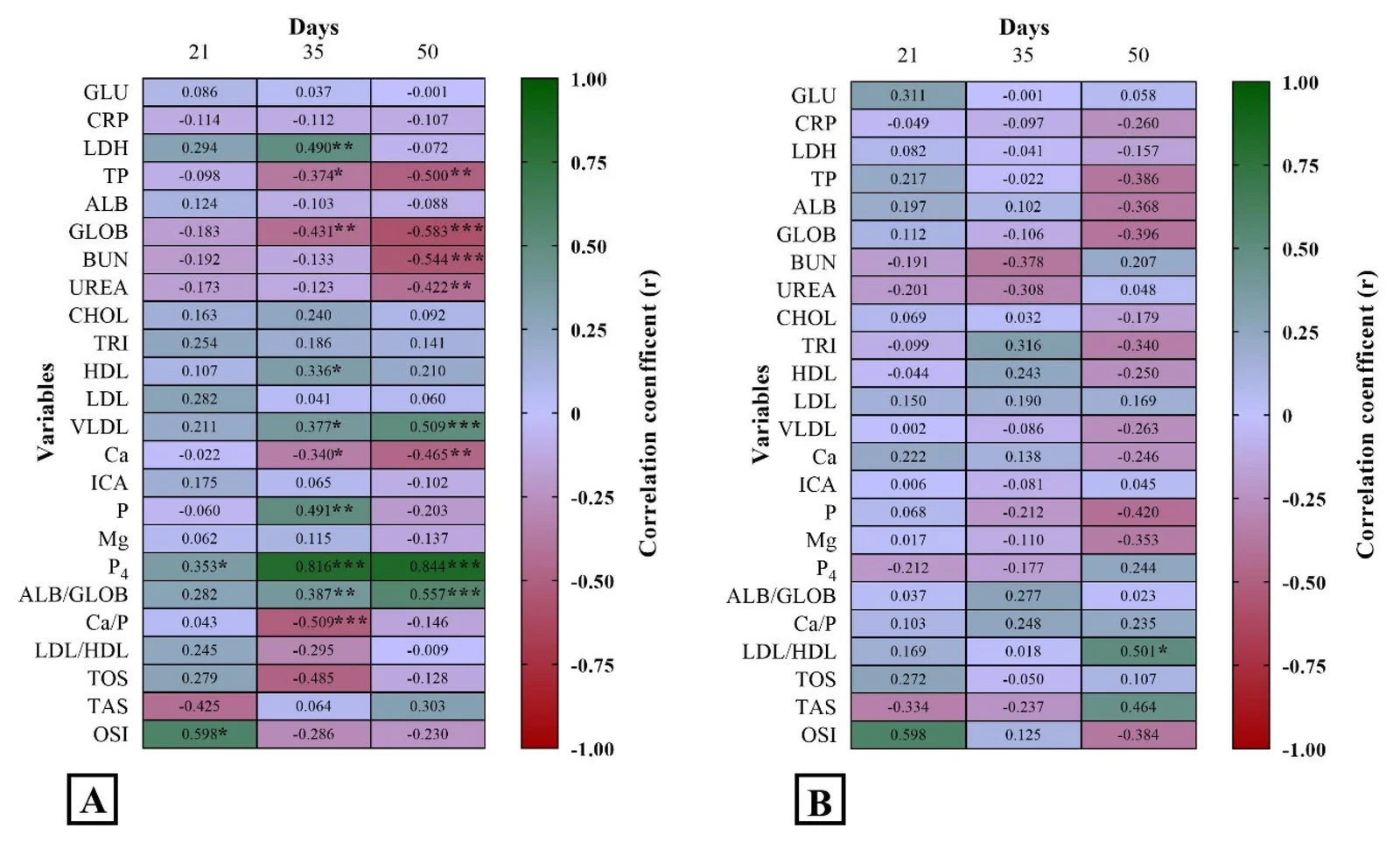
Correlations between serum pregnancy-associated glycoprotein (PAG) concentrations and maternal biochemical, metabolic, mineral, and oxidative stress variables at days 21, 35, and 50 after mating. Heatmaps show Pearson’s correlation coefficients (r) calculated for (**A**) all ewes and (**B**) pregnant ewes only. Asterisks indicate statistical significance: *p* < 0.05 (*), *p* < 0.01 (**), and *p* < 0.001 (***).

**Figure 9:**
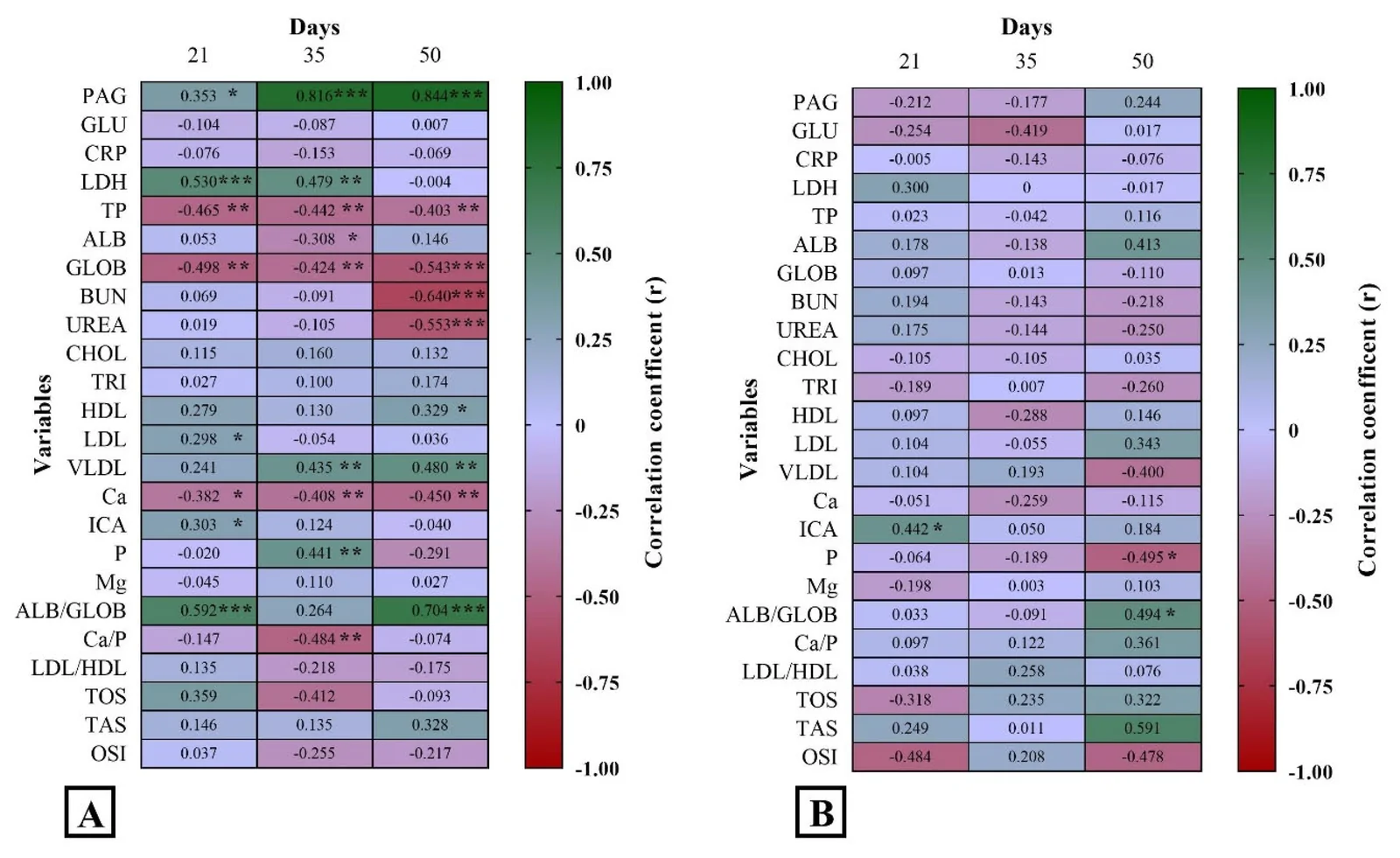
Correlations between serum progesterone (P_4_) concentrations and maternal biochemical, metabolic, mineral, and oxidative stress variables at days 21, 35, and 50 after mating. Heatmaps show Pearson’s correlation coefficients (r) calculated for (**A**) all ewes and (**B**) pregnant ewes only. Asterisks indicate statistical significance: *p* < 0.05 (*), *p* < 0.01 (**), and *p* < 0.001 (***).

**Figure 10:**
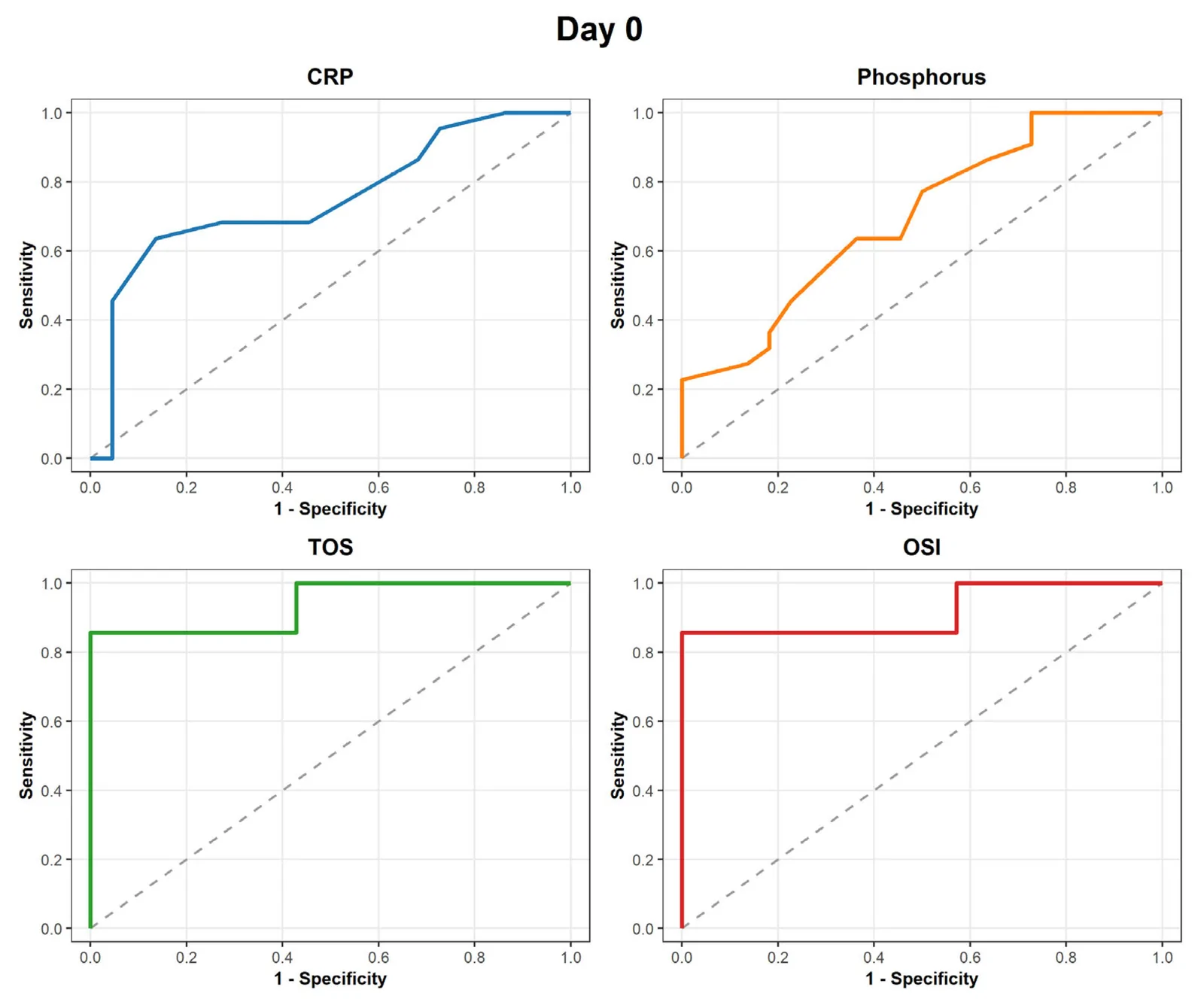
Receiver operating characteristic (ROC) curves for the discrimination of subsequent maintained pregnancy outcome using maternal biochemical and oxidative stress variables on D0. ROC curves are shown for C-reactive protein (CRP), phosphorus (P), total oxidant status (TOS), and oxidative stress index (OSI). The dashed diagonal line represents the line of no discrimination (area under the curve [AUC] = 0.50).

**Figure 11:**
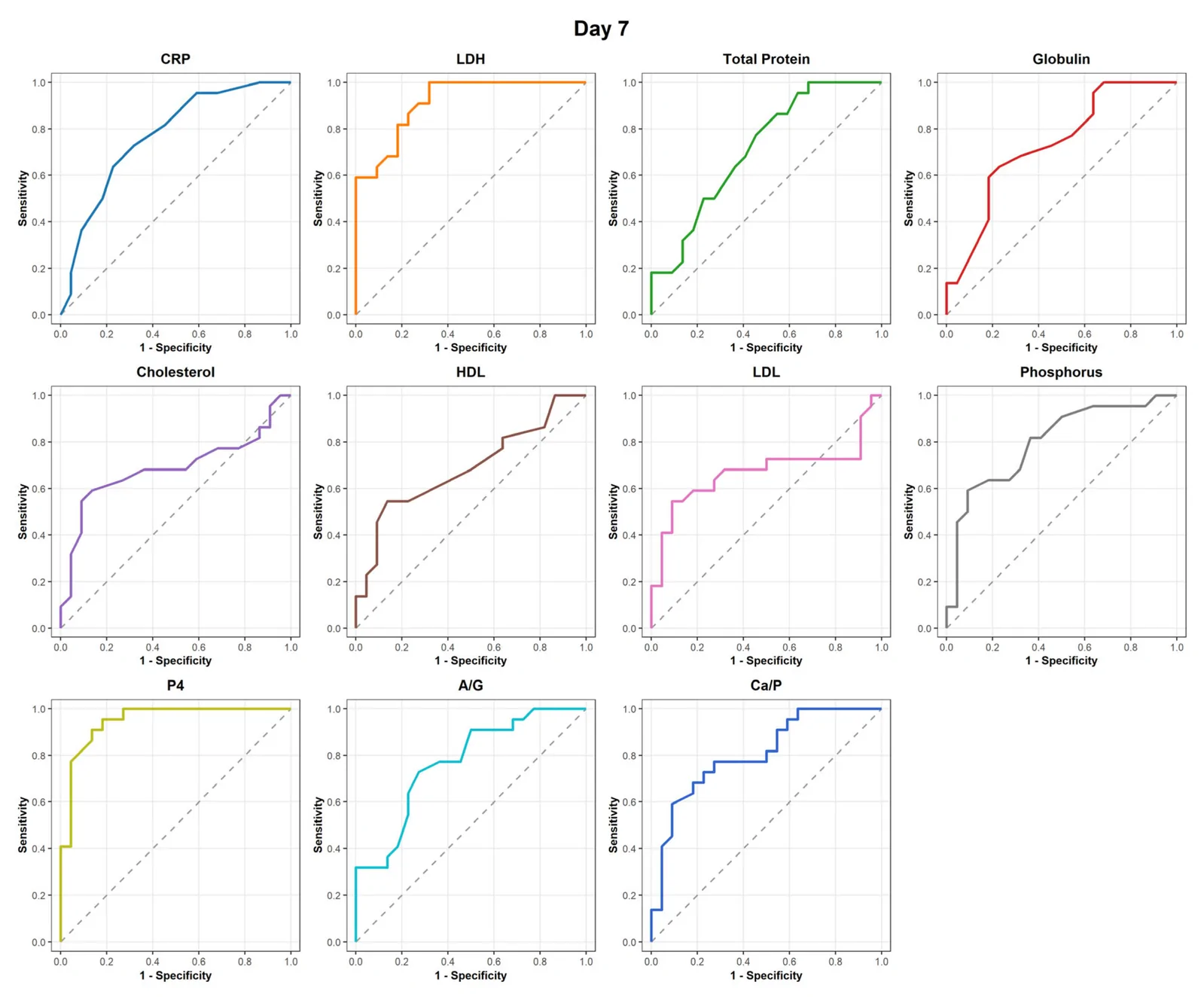
Receiver operating characteristic (ROC) curves for the discrimination of pregnant and non-pregnant ewes using maternal biochemical and hormonal variables on D7. ROC curves are shown for C-reactive protein (CRP), lactate dehydrogenase (LDH), total protein, globulin, cholesterol, high-density lipoprotein cholesterol (HDL-C), low-density lipoprotein cholesterol (LDL-C), phosphorus (P), progesterone (P_4_), albumin-to-globulin ratio (A/G), and calcium-to-phosphorus ratio (Ca/P). The dashed diagonal line represents the line of no discrimination (area under the curve [AUC] = 0.50).

**Figure 12:**
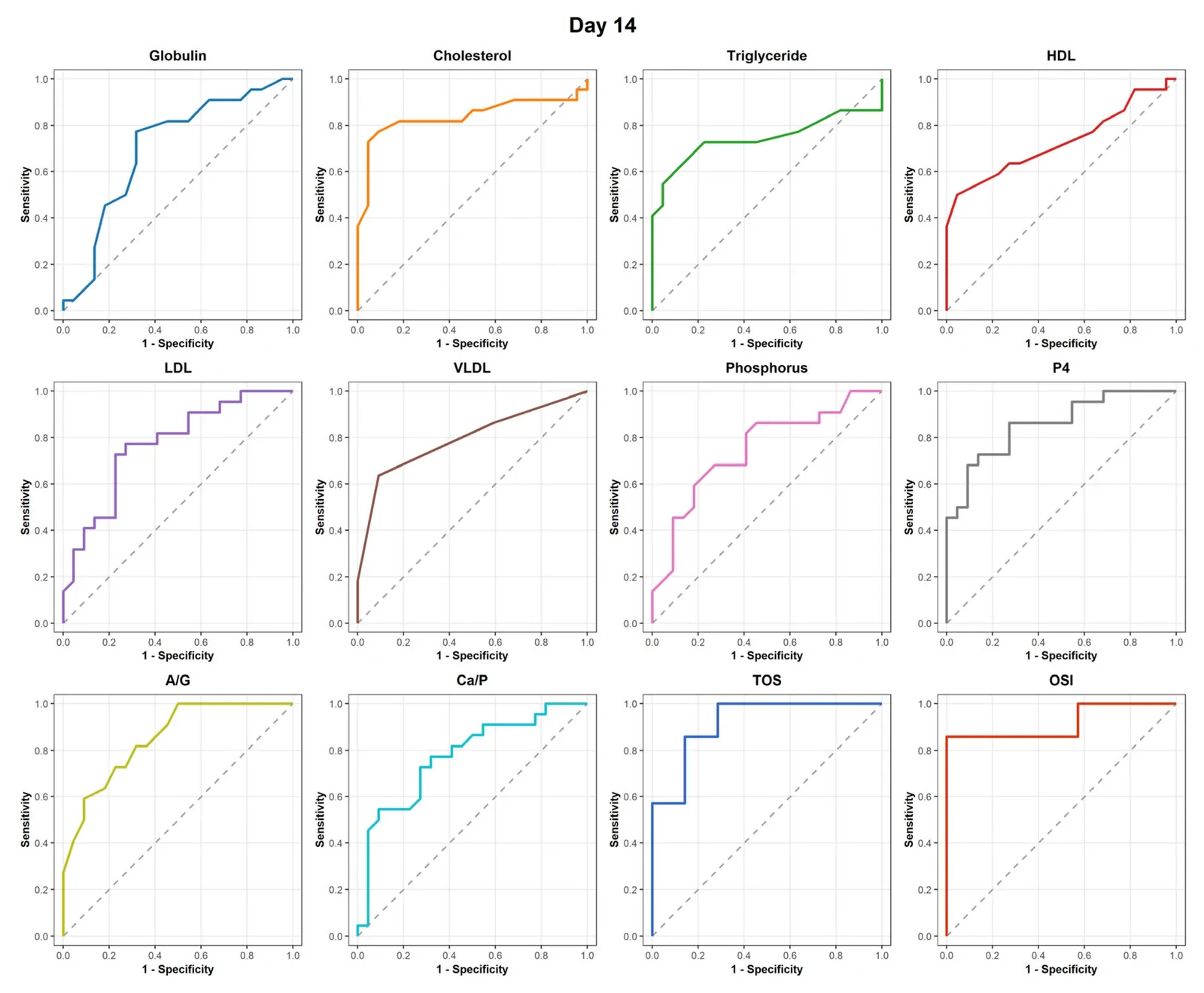
Receiver operating characteristic (ROC) curves for the discrimination of pregnant and non-pregnant ewes using maternal biochemical, hormonal, and oxidative stress variables on D14. ROC curves are shown for globulin, cholesterol, triglycerides, high-density lipoprotein cholesterol (HDL-C), low-density lipoprotein cholesterol (LDL-C), very-low-density lipoprotein cholesterol (VLDL), phosphorus (P), progesterone (P_4_), albumin-to-globulin ratio (A/G), calcium-to-phosphorus ratio (Ca/P), total oxidant status (TOS), and oxidative stress index (OSI). The dashed diagonal line represents the line of no discrimination (area under the curve [AUC] = 0.50).

**Figure 13:**
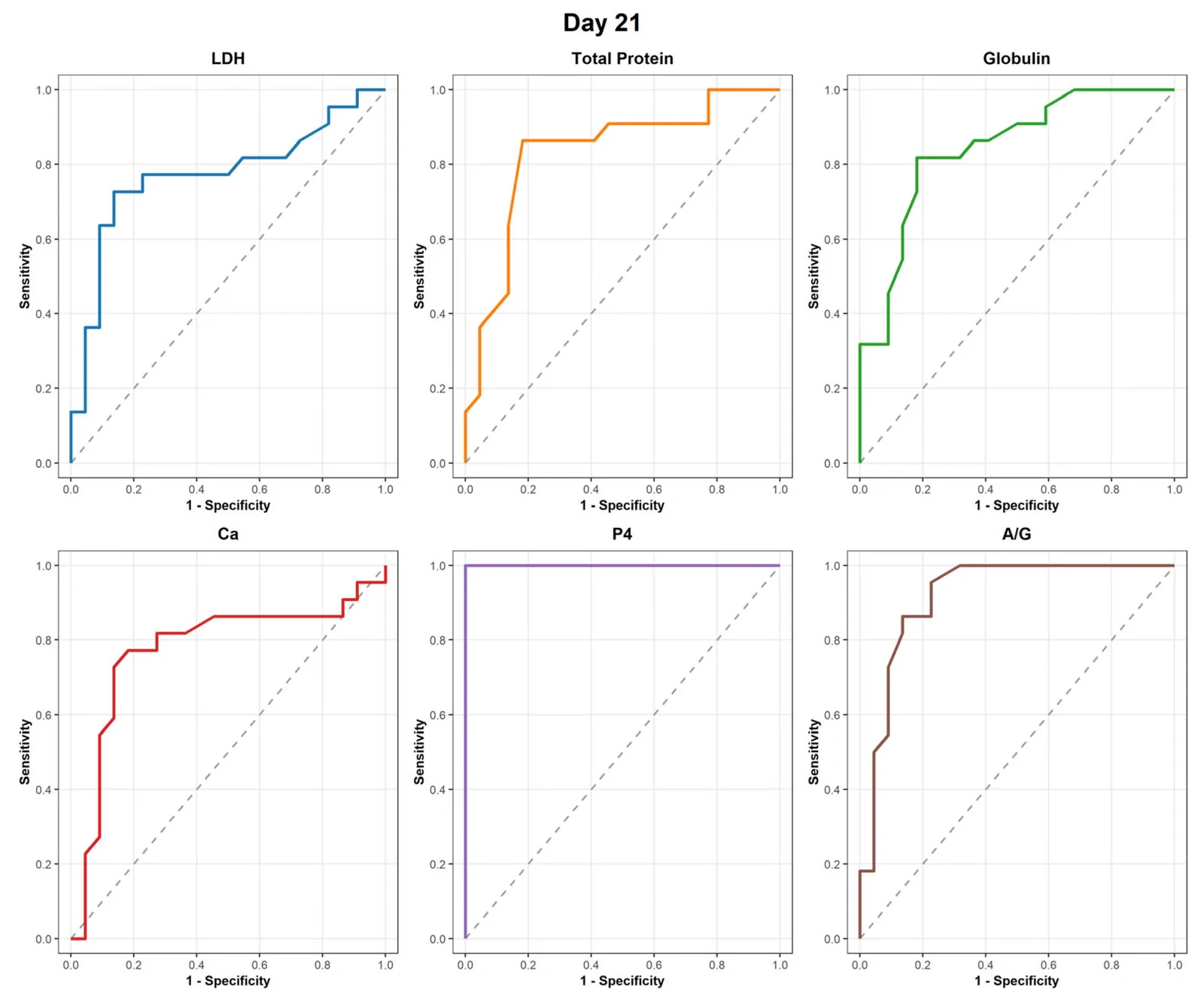
Receiver operating characteristic (ROC) curves for the discrimination of pregnant and non-pregnant ewes using maternal biochemical and hormonal variables on D21. ROC curves are shown for lactate dehydrogenase (LDH), total protein, globulin, calcium (Ca), progesterone (P_4_), and albumin-to-globulin ratio (A/G). The dashed diagonal line represents the line of no discrimination (area under the curve [AUC] = 0.50).

**Figure 14:**
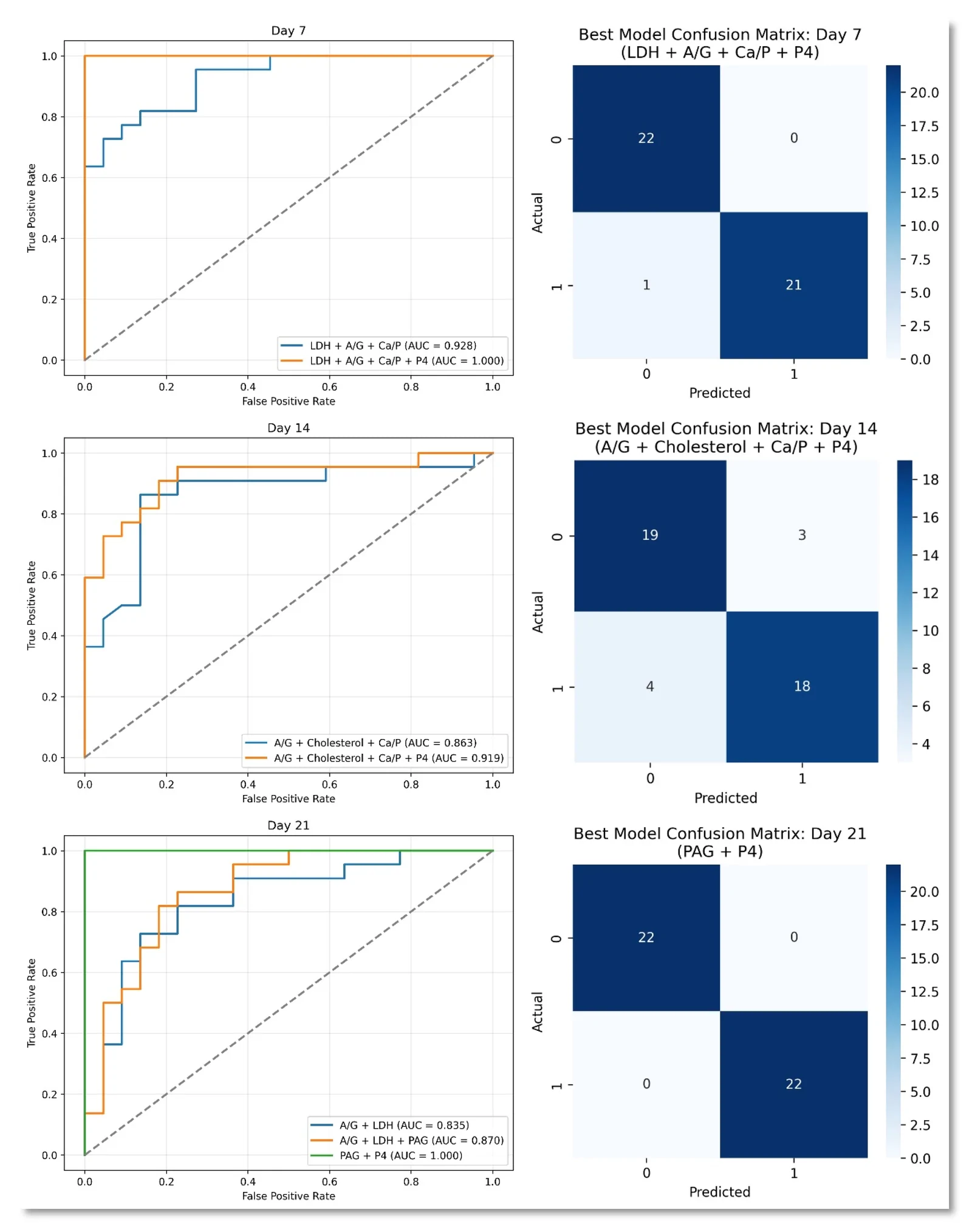
Receiver operating characteristic (ROC) curves and confusion matrices for the discrimination of pregnant and non-pregnant ewes using multivariable models on D7, D14, and D21. Models are shown for LDH + A/G + Ca/P with and without progesterone (P_4_) on D7, A/G + cholesterol + Ca/P with and without P_4_ on D14, and A/G + LDH, A/G + LDH + pregnancy-associated glycoprotein (PAG), and PAG + P_4_ on D21. Confusion matrices show the classification performance of the best-performing model for each day. The dashed diagonal line represents the line of no discrimination (area under the curve [AUC] = 0.50).

## Data Availability

The original contributions presented in this study are included in the article. Further inquiries can be directed at the corresponding author.

## Supplementary Materials

The following supporting information can be downloaded at https://www.mdpi.com/article/10.3390/vetsci13101003/s1. Table S1: Serum protein-related variables from D0 to D50; Table S2: Serum lipid-related variables from D0 to D50; Table S3: Serum mineral-related variables from D0 to D50; Table S4: Glucose, lactate dehydrogenase (LDH), and C-reactive protein (CRP) from D0 to D50; Table S5: Oxidative status from D0 to D50; Table S6: Serum progesterone (P_4_) and pregnancy-associated glycoprotein (PAG) concentrations from D0 to D50; Table S7: Analytical performance of serum biochemical assays; Dataset 1: Correlation matrices among pregnancy markers and maternal biochemical variables in all ewes and stratified by pregnancy status at D21, D35, and D50, together with receiver operating characteristic (ROC) performance analyses of individual variables and multivariable models.

## Abbreviations

The following abbreviations are used in this manuscript:

A/G	Albumin-to-globulin ratio
AIC	Akaike information criterion
ATP	Adenosine triphosphate
AUC	Area under the curve
BCS	Body condition score
BUN	Blood urea nitrogen
BW	Body weight
Ca/P	Calcium-to-phosphorus ratio
CL	Corpus luteum
CRP	C-reactive protein
eCG	Equine chorionic gonadotropin
ELISA	Enzyme-linked immunosorbent assay
GLU	Glucose
HDL-C	High-density lipoprotein cholesterol
ICA	Ionized calcium
IFN-τ	Interferon tau
LDH	Lactate dehydrogenase
LDHA	Lactate dehydrogenase A
LDHB	Lactate dehydrogenase B
LDL-C	Low-density lipoprotein cholesterol
LDL/HDL	Low-density lipoprotein-to-high-density lipoprotein ratio
MAP	Medroxyprogesterone acetate
MDA	Malondialdehyde
NO	Nitric oxide
O.D.	Optical density
OSI	Oxidative stress index
P_4_	Progesterone
PAG	Pregnancy-associated glycoproteins
PGF_2_α	Prostaglandin F2 alpha
RIA	Radioimmunoassay
ROC	Receiver operating characteristic
SAA	Serum amyloid A
S-N	Sample-negative control
TAS	Total antioxidant status
TOS	Total oxidant status
TP	Total protein
VLDL	Very-low-density lipoprotein
Q-Q	Quantile–quantile
