# Supplementary material for: Maternal Metabolic Profiles, Their Relationships with Progesterone and Pregnancy-Associated Glycoprotein (PAG), and Biomarker Potential During Early Pregnancy in Lactating Ewes
Source: Vet Sci. 2026 Sep 23;13(10):1003. doi: 10.3390/vetsci13101003 (PMC13709780; doi:10.3390/vetsci13101003)
Supplement: Supplementary file 1 [file vetsci-13-01003-s001.zip › Clean Revise-Supplementary Tables.pdf]

SUPPLEMENTARY TABLES

Table S1. Longitudinal changes in serum protein-related variables in pregnant and non-pregnant ewes from D0 to D50.

Parameter	Group	Day						*LS Mean (Group)*	**P*-value		
		**D0**	**D7**	**D14**	**D21**	**D35**	**D50**		**G**	**D**	**G × D**
**TP (g/dL)**	**Non-pregnant**	6.195 ± 0.115 ^{ab}	6.664 ± 0.163 $^{a, A}$	6.432 ± 0.182 ^{ab}	6.823 ± 0.178 $^{a, A}$	5.877 ± 0.168 $^{b, A}$	6.791± 0.194 $^{a, A}$	**6.464 ± 0.074**	**<0.001**	**<0.001**	**0.026**
	**Pregnant**	6.136 ± 0.108 ^{a}	6.032 ± 0.120 $^{a, B}$	6.132 ± 0.135 ^{a}	5.827 ± 0.168 $^{ab, B}$	5.273 ± 0.141$^{b, B}$	5.918 ± 0.141 $^{a, B}$	**5.886 ± 0.061**			
	LS Mean (Day)	**6.166 ± 0.078**	**6.348 ± 0.111**	**6.282 ± 0.114**	**6.325 ± 0.143**	**5.575 ± 0.118**	**6.355 ± 0.136**				
	****P*-value**	0.710	**0.003**	0.194	**<0.001**	**0.009**	**<0.001**				
**Albumin (g/dL)**	**Non-pregnant**	2.068± 0.034	2.105 ± 0.048	2.091 ± 0.059	2.273 ± 0.048	2.123 ± 0.049	2.282 ± 0.073	2.157 ± 0.023	**0.710**	**0.001**	**0.513**
	**Pregnant**	2.032 ± 0.037	2.145 ± 0.044	2.218 ± 0.047	2.268 ± 0.053	2.050 ± 0.064	2.295 ± 0.065	2.168 ± 0.023			
	LS Mean (Day)	**2.050 ± 0.025 ^{c}**	**2.125 ± 0.032 ^{bc}**	**2.155± 0.039 ^{abc}**	**2.270 ± 0.035 ^{ab}**	**2.086± 0.040 ^{c}**	**2.289 ± 0.048 ^{a}**				
	****P*-value**	0.475	0.534	0.099	0.950	0.372	0.890				
**Globulin (g/dL)**	**Non-pregnant**	4.127± 0.111 ^{ab}	4.514 ± 0.149 $^{a, A}$	4.295± 0.129 $^{a, A}$	4.595 ± 0.163 $^{a, A}$	3.755 ± 0.142 $^{b, A}$	4.509 ± 0.140 $^{a, A}$	**4.299 ± 0.61**	**<0.001**	**<0.001**	**0.004**
	**Pregnant**	4.095 ± 0.112 ^{a}	3.886 ± 0.110 $^{a, B}$	3.905± 0.105 $^{a, B}$	3.605 ± 0.134 $^{ab, B}$	3.223 ± 0.091 $^{b, B}$	3.668 ± 0.110 $^{ab, B}$	**3.730 ± 0.051**			
	LS Mean (Day)	**4.111± 0.078**	**4.200 ± 0.103**	**4.100 ± 0.087**	**3.489 ± 0.129**	**3.489 ± 0.093**	**4.089 ± 0.109**				
	****P*-value**	0.841	**0.002**	**0.024**	**<0.001**	**0.003**	**<0.001**				
**A/G (ratio)**	**Non-pregnant**	0.509 ± 0.016 ^{b}	0.473 ± 0.015 $^{b, B}$	0.490 ± 0.011$^{b, B}$	0.505 ± 0.016 $^{b, B}$	0.579 ± 0.020 $^{a, B}$	0.510 ± 0.014 $^{b, B}$	**0.511 ± 0.007**	**<0.001**	**<0.001**	**0.001**
	**Pregnant**	0.505± 0.018 ^{b}	0.563 ± 0.023 $^{b, A}$	0.574 ± 0.013 $^{ab, A}$	0.640 ± 0.017 $^{a, A}$	0.640 ± 0.015 $^{a, A}$	0.633 ± 0.017 $^{a, A}$	**0.592 ± 0.008**			
	LS Mean (Day)	**0.507 ± 0.12**	**0.518 ± 0.015**	**0.532 ± 0.011**	**0.572 ± 0.016**	**0.609 ± 0.013**	**0.571 ± 0.015**				
	****P*-value**	0.887	**0.002**	**<0.001**	**<0.001**	**0.016**	**<0.001**				
**BUN (mg/dL)**	**Non-pregnant**	16.864 ± 0.848 ^{a}	12.591 ± 0.454 ^{b}	11.955 ± 0.381 ^{b}	16.000 ± 0.827 ^{a}	7.682 ± 0.266 ^{c}	17.545± 0.686 $^{a, A}$	13.773 ± 0.389	**0.087**	**<0.001**	**<0.001**
	**Pregnant**	17.364 ± 1.438 ^{a}	12.545 ± 0.464 ^{b}	12.545 ± 0.320 ^{b}	16.091 ± 0.811 ^{a}	7.591 ± 0.393 ^{c}	12.364 ± 0.564 $^{b, B}$	13.083 ± 0.411			
	LS Mean (Day)	**17.114 ± 0.826**	**12.568 ± 0.321**	**12.250 ± 0.250**	**16.045 ± 0.572**	**7.636 ± 0.235**	**14.955 ± 0.591**				
	****P*-value**	0.731	0.945	0.242	0.938	0.849	**<0.001**				
**Urea (mg/dL)**	**Non-pregnant**	34.445 ± 1.544 ^{a}	27.332 ± 0.943 ^{b}	26.068 ± 0.890 ^{b}	35.436 ± 1.650 ^{a}	16.014 ± 0.577 ^{c}	35.091 ± 2.030 $^{a, A}$	**29.064 ± 0.817**	**0.031**	**<0.001**	**0.001**
	**Pregnant**	34.491 ± 1.396 ^{a}	26.259 ± 0.812 ^{b}	27.473 ± 0.735 ^{b}	34.805 ± 1.760 ^{a}	15.700 ± 0.809 ^{c}	25.964 ± 1.311^{b,B}	**27.448 ± 0.737**			
	LS Mean (Day)	**34.468 ± 1.029**	**26.795 ± 0.620**	**26.770 ± 0.580**	**35.120 ± 1.193**	**15.857 ± 0.492**	**30.527± 1.382**				
	****P*-value**	0.983	0.394	0.231	0.795	0.754	**<0.001**				

**Values are presented as least-squares means (LS means) ± SE. G, main effect of pregnancy group; D, main effect of sampling day; and G × D, pregnancy group-by-day interaction. For variables with a significant G × D interaction, different lowercase letters ($^{a, b, c}$) indicate significant differences among sampling days within the same group, whereas different uppercase letters ($^{A, B}$) indicate significant differences between the non-pregnant and pregnant groups within the same sampling day (P<0.05). Means sharing the same letter or without letters do not differ significantly. The P values presented in the **P-value row below each variable represents the pairwise comparison between the non-pregnant and pregnant groups at each sampling day. When the G × D interaction was not significant, letters accompanying the overall LS means for group or day indicate significant main-effect differences (P<0.05). Abbreviations: TP, total protein; A, albumin; G, globulin; A/G, albumin-to-globulin ratio; BUN, blood urea nitrogen.*

Table S2. Longitudinal changes in serum lipid-related variables in pregnant and non-pregnant ewes from D0 to D50.

Parameter	Group	Day						*LS Mean (Group)*	**P*-value		
		D0	D7	D14	D21	D35	D50		G	D	G × D
Cholesterol (mg/dL)	Non-pregnant	38.636 ± 1.524	32.136 ± 1.362	37.636 ± 1.423	43.955 ± 2.337	40.182 ± 1.544	46.000 ± 1.654	**39.758 ± 0.776 ^{B}**	**<0.001**	**<0.001**	**0.136**
	Pregnant	38.636 ± 1.570	38.227 ± 2.168	48.000 ± 2.146	48.091 ± 2.875	44.727 ± 2.228	48.091 ± 1.699	**44.295 ± 0.942 ^{A}**			
	LS Mean (Day)	**38.636 ± 1.081 ^{bc}**	**35.182 ±1.348 ^{c}**	**42.818± 1.498 ^{ab}**	**46.023 ±1.858 ^{a}**	**42.455 ± 1.384 ^{ab}**	**47.045 ± 1.183 ^{a}**				
	***P*-value	**1.000**	**0.023**	**<0.001**	**0.271**	**0.102**	**0.383**				
Triglycerides (mg/dL)	Non-pregnant	13.000 ± 0.712	12.727 ± 0.782	13.500 ± 0.450	14.727 ± 0.972	12.591 ± 0.491	23.636 ± 0.941	**15.030 ± 0.456 ^{B}**	**<0.001**	**<0.001**	**0.139**
	Pregnant	13.227 ± 0.868	13.727 ± 1.030	17.318 ± 1.068	15.545 ± 0.894	13.227 ± 0.606	27.818 ± 1.817	**16.811 ± 0.632 ^{A}**			
	LS Mean (Day)	**13.114 ± 0.555 ^{b}**	**13.227 ± 0.644 ^{b}**	**15.409 ± 0.642 ^{b}**	**15.136 ± 0.656 ^{b}**	**12.909 ± 0.389 ^{b}**	**25.727 ± 1.060 ^{a}**				
	***P*-value	**0.841**	**0.444**	**0.003**	**0.539**	**0.419**	**0.049**				
HDL-C (mg/dL)	Non-pregnant	19.273 ± 0.684	19.227 ± 0.753	21.045 ± 0.725	23.091 ± 0.750	19.455 ± 0.623	21.545 ± 0.761	**20.606± 0.313 ^{B}**	**<0.001**	**<0.001**	**0.586**
	Pregnant	20.318 ± 0.799	22.727 ± 1.153	25.591 ± 1.196	25.864 ± 1.320	21.955 ± 1.078	24.636± 1.156	**23.515± 0.485 ^{A}**			
	LS Mean (Day)	**19.64 ± 0.526 ^{c}**	**20.977 ± 0.731 ^{bc}**	**23.318 ± 0.773 ^{ab}**	**24.477 ± 0.779 ^{a}**	**20.705 ± 0.644 ^{bc}**	**23.091 ± 0.723 ^{ab}**				
	***P*-value	**0.326**	**0.015**	**0.003**	**0.077**	**0.053**	**0.032**				
LDL-C (mg/dL)	Non-pregnant	17.373 ± 1.174 ^{ab}	9.809 ± 0.790 $^{c, B}$	14.300 ± 0.956 ^{bc,B}	21.236 ± 0.997 $^{a.B}$	19.395 ± 0.952 ^{a}	20.214 ± 1.262 ^{a}	**17.05 ± 0.538**	**0.008**	**<0.001**	**0.036**
	Pregnant	16.268 ± 1.346 ^{bc}	12.800 ± 1.221 ^{c,A}	19.400 ± 1.102 $^{b.A}$	24.755 ± 1.489 $^{a.A}$	19.382± 1.231^{b}	20.068± 0.741 ^{ab}	**18.779 ± 0.581**			
	LS Mean (Day)	**16.820 ± 0.887**	**11.305 ± 0.754**	**16.850 ± 0.819**	**22.965 ± 0.925**	**19.389 ± 0.769**	**20.141 ± 0.723**				
	***P*-value	**0.540**	**0.047**	**0.001**	**0.057**	**0.993**	**0.921**				
VLDL (mg/dL)	Non-pregnant	2.545 ± 0.143 ^{b}	2.364 ± 0.124 ^{b,B}	2.682 ± 0.138 ^{b,B}	2.727 ± 0.176 ^{b}	2.409 ± 0.126 ^{b,B}	4.273 ± 0.210 ^{a,B}	**2.83± 0.085**	**<0.001**	**<0.001**	**<0.001**
	Pregnant	2.591± 0.157 ^{c}	2.909 ± 0.227 ^{c,A}	3.682± 0.202 ^{b,A}	3.136 ± 0.201^{bc}	3.000 ± 0.147 ^{bc,A}	6.000±0.237 ^{a,A}	**3.55± 0.127**			
	LS Mean (Day)	**2.568 ± 0.105**	**2.636 ± 0.134 ^{a}**	**3.182 ± 0.143**	**2.932 ± 0.136**	**2.705 ± 0.106**	**5.136±0.205**				
	***P*-value	**0.831**	**0.043**	**<0.001**	**0.133**	**0.004**	**<0.001**				

**Values are presented as least-squares means (LS means) ± SE. G, main effect of pregnancy group; D, main effect of sampling day; and G × D, pregnancy group-by-day interaction. For variables with a significant G × D interaction, different lowercase letters ($^{a, b, c}$) indicate significant differences among sampling days within the same group, whereas different uppercase letters ($^{A, B}$) indicate significant differences between the non-pregnant and pregnant groups within the same sampling day ($P<0.05$). Means sharing the same letter or without letters do not differ significantly. The P values presented in the **P-value row below each variable represents pairwise comparisons between the non-pregnant and pregnant groups at each sampling day. When the G × D interaction was not significant, letters accompanying the overall LS means for group or day indicate significant main-effect differences ($P<0.05$). Abbreviations: HDL-C, high-density lipoprotein cholesterol; LDL-C, low-density lipoprotein cholesterol; VLDL, very-low-density lipoprotein.*

Table S3. Longitudinal changes in serum mineral-related variables in pregnant and non-pregnant ewes from D0 to D50.

Parameter	Group	Day						*LS Mean (Group)*	**P*-value		
		D0	D7	D14	D21	D35	D50		G	D	G × D
Ca (mg/dL)	Non-pregnant	8.414 ± 0.105	9.005 ± 0.172	8.991 ± 0.207	9.105 ± 0.181	8.241 ± 0.193	8.818± 0.179	**8.762 ± 0.076 ^{A}**			
	Pregnant	8.350 ± 0.144	8.664 ± 0.153	8.727 ± 0.187	8.277 ± 0.216	7.641 ± 0.119	8.018 ± 0.149	**8.280 ± 0.073 ^{B}**	**<0.001**	**<0.001**	**0.149**
	LS Mean (Day)	**8.382 ± 0.088 ^{ab}**	**8.834 ± 0.117 ^{a}**	**8.859 ± 0.139 ^{a}**	**8.691 ± 0.153 ^{a}**	**7.941 ± 0.121 ^{b}**	**8.41 ± 0.130 ^{ab}**				
	*****P*-value**	**0.723**	**0.146**	**0.350**	**0.005**	**0.012**	**0.001**				
P (mg/dL)	Non-pregnant	3.659 ± 0.109 ^{ab,B}	2.950 ± 0.114 ^{c,B}	3.832 ± 0.142 ^{a,B}	3.091 ± 0.143 ^{bc}	3.173 ± 0.103 ^{bc,B}	4.105 ± 0.131^{a}	**3.468 ± 0.062**			
	Pregnant	4.068 ± 0.130 ^{ab,A}	3.614 ± 0.129 ^{b,A}	4.482 ± 0.165 ^{a,A}	3.091 ± 0.131^{c}	3.900 ± 0.129 $^{b, A}$	3.914± 0.099 ^{b}	**3.845 ± 0.065**	**<0.001**	**<0.001**	**0.001**
	LS Mean (Day)	**3.864 ± 0.090**	**3.282± 0.099**	**4.157± 0.118**	**3.091± 0.096**	**3.536 ± 0.098**	**4.009± 0.082**				
	*****P*-value**	**0.021**	**<0.001**	**0.005**	**1.000**	**<0.001**	**0.252**				
Mg (mg/dL)	Non-pregnant	2.423 ± 0.094	3.028 ± 0.132	2.963± 0.109	2.495 ± 0.056	2.382 ± 0.040	2.401 ± 0.039	2.615 ± 0.042			
	Pregnant	2.470 ± 0.118	2.956 ± 0.134	3.021± 0.134	2.505 ± 0.054	2.445 ± 0.045	2.393 ± 0.050	2.632 ± 0.045	**0.756**	**<0.001**	**0.978**
	LS Mean (Day)	**2.446 ± 0.074 ^{b}**	**2.992± 0.093 ^{a}**	**2.992 ± 0.085 ^{a}**	**2.500 ± 0.038 ^{b}**	**2.414 ± 0.030 ^{b}**	**2.397± 0.031 ^{b}**				
	*****P*-value**	**0.759**	**0.706**	**0.736**	**0.893**	**0.300**	**0.903**				
Ca/P (ratio)	Non-pregnant	2.337 ± 0.063 ^{bc}	3.146 ± 0.134 ^{a,A}	2.400 ± 0.089 ^{bc,A}	3.085 ± 0.158 ^{a,A}	2.671 ± 0.128 ^{b,A}	2.194 ± 0.082 ^{c}	**2.639 ± 0.56**			
	Pregnant	2.089 ± 0.064 ^{bc}	2.462 ± 0.097 ^{ab,B}	1.994 ± 0.075 ^{c,B}	2.795 ± 0.153 ^{a,B}	2.005 ± 0.075 ^{c,B}	2.072 ± 0.059 ^{bc}	**2.236 ± 0.45**	**<0.001**	**<0.001**	**0.035**
	LS Mean (Day)	**2.213± 0.48**	**2.804 ± 0.097**	**2.197 ± 0.65**	**2.940 ± 0.111**	**2.338 ± 0.89**	**2.133± 0.051**				
	*****P*-value**	**0.009**	**<0.001**	**0.001**	**0.194**	**<0.001**	**0.232**				

**Values are presented as least-squares means (LS means) ± SE. G, main effect of pregnancy group; D, main effect of sampling day; and G × D, pregnancy group-by-day interaction. For variables with a significant G × D interaction, different lowercase letters ($^{a, b, c}$) indicate significant differences among sampling days within the same group, whereas different uppercase letters ($^{A, B}$) indicate significant differences between the non-pregnant and pregnant groups within the same sampling day (P<0.05). Means sharing the same letter or without letters do not differ significantly. The P values presented in the **P-value row below each variable represent pairwise comparisons between the non-pregnant and pregnant groups at each sampling day. When the G × D interaction was not significant, letters accompanying the overall LS means for group or day indicate significant main-effect differences (P<0.05). Abbreviations: Ca, calcium; P, phosphorus; Mg, magnesium; Ca/P, calcium-to-phosphorus ratio.*

Table S4. Longitudinal changes in glucose, lactate dehydrogenase (LDH), and C-reactive protein (CRP) in pregnant and non-pregnant ewes from D0 to D50.

Parameter	Group	Day						*LS Mean (Group)*	**P*-value		
		**D0**	**D7**	**D14**	**D21**	**D35**	**D50**		**G**	**D**	**G × D**
**Glucose (mg/dL)**	**Non-pregnant**	80.636 ± 4.882	71.591 ± 3.110	69.318 ± 4.045	87.909 ± 2.660	85.500 ± 2.602	76.68 ± 1.752	78.606± 1.462			
	**Pregnant**	78.636 ± 2.590	71.136 ± 1.887	70.500 ± 2.290	87.182 ± 2.296	86.318 ± 2.108	76.68 ± 1.029	78.409± 1.015	**0.903**	**<0.001**	**0.995**
	LS Mean (Day)	**79.636 ± 2.735 ^{ab}**	**71.364 ± 1.798 ^{c}**	**69.909 ± 2.299 ^{c}**	**87.545 ± 1.737 ^{a}**	**85.909 ± 1.656 ^{a}**	**76.68 ± 1.004 ^{bc}**				
	****P*-value**	**0.720**	**0.901**	**0.801**	**0.837**	**0.808**	**1.000**				
**LDH (U/L)**	**Non-pregnant**	262.432±13.766 ^{b}	294.227 ± 8.088 ^{b,B}	362.500 ± 18.477 ^{a,B}	207.136±9.800 ^{c,B}	200.636±8.411 ^{c,B}	202.182± 8.286^{c}	**254.852±7.013**			
	**Pregnant**	262.045 ± 6.135 ^{b}	412.727 ± 17.792 ^{a,A}	405.818 ± 19.348 ^{a,A}	270.727±14.942 ^{b,A}	250.818 ± 9.475 ^{b,A}	203.545± 10.881^{c}	**300.947±8.901**	**<0.001**	**<0.001**	**<0.001**
	LS Mean (Day)	**262.239± 7.447**	**353.477± 13.225**	**384.159 ± 13.627**	**238.932 ± 10.074**	**225.727 ± 7.337**	**202.864 ± 6.759**				
	****P*-value**	**0.980**	**<0.001**	**0.113**	**0.001**	**<0.001**	**0.921**				
**CRP (mg/dL)**	**Non-pregnant**	0.538± 0.005	0.547± 0.007	0.536 ± 0.006	0.540± 0.007	0.535± 0.007	0.541± 0.007	**0.540 ± 0.003 ^{A}**			
	**Pregnant**	0.516± 0.005	0.518± 0.005	0.524± 0.006	0.535± 0.005	0.529± 0.006	0.538± 0.006	**0.527 ± 0.002^{B}**	**<0.001**	**0.337**	**0.188**
	LS Mean (Day)	0.527± 0.004	0.533± 0.005	0.530± 0.004	0.537± 0.004	0.532± 0.005	0.540± 0.005				
	****P*-value**	**0.002**	**0.001**	**0.176**	**0.589**	**0.514**	**0.702**				

**Values are presented as least-squares means (LS means) ± SE. G, main effect of pregnancy group; D, main effect of sampling day; and G × D, pregnancy group-by-day interaction. For variables with a significant G × D interaction, different lowercase letters ($^{a, b, c}$) indicate significant differences among sampling days within the same group, whereas different uppercase letters ($^{A, B}$) indicate significant differences between the non-pregnant and pregnant groups within the same sampling day ($P < 0.05$). Means sharing the same letter or without letters do not differ significantly. The P values presented in the **P-value row below each variable represents pairwise comparisons between the non-pregnant and pregnant groups at each sampling day. When the G × D interaction was not significant, letters accompanying the overall LS means for group or day indicate significant main-effect differences ($P < 0.05$). Abbreviations: LDH, Lactate dehydrogenase, CRP; C-reactive protein.*

Table S5. Longitudinal changes in systemic oxidative status in pregnant and non-pregnant ewes from D0 to D50.

Parameter	Group	Day						*LS Mean (Group)*	****P*-value**		
		**D0**	**D7**	**D14**	**D21**	**D35**	**D50**		**G**	**D**	**G × D**
**TOS (µmol H_2O_2 Equiv./L)**	**Non-pregnant**	26.534 ± 1.202 ^{A}	23.393 ± 4.547	30.933 ± 3.471 ^{A}	26.087 ± 0.910	30.923± 1.279	32.636± 5.490	**28.418± 1.375**			
	**Pregnant**	16.159 ± 2.238 ^{b,B}	30.277 ± 2.995 ^{a}	18.503 ± 1.350 ^{b,B}	29.609 ± 1.518 ^{a}	27.786 ± 0.824^{a}	29.954± 1.326^{a}	**25.381± 1.148**	**0.055**	**0.008**	**0.003**
	LS Mean (Day)	**21.346 ± 1.887**	**26.835 ± 2.784**	**24.718 ± 2.485**	**27.848± 0.980**	**29.354± 0.851**	**31.295± 2.738**				
	*****P*-value**	**0.003**	**0.234**	**0.011**	**0.075**	**0.066**	**0.650**				
**TAS (mmol Trolox Equiv./L)**	**Non-pregnant**	628.107± 19.685	672.994± 30.912	680.449± 51.950	717.959± 60.928	624.951± 44.430	654.284± 60.858	663.124± 18.779			
	**Pregnant**	675.993± 25.987	772.202± 62.172	687.007± 57.484	743.885± 77.678	661.784± 55.876	735.894± 112.274	712.794± 27.592	**0.155**	**0.616**	**0.973**
	LS Mean (Day)	652.050± 17.011	722.598± 36.080	683.728± 37.231	730.922± 47.561	661.784± 34.672	695.089± 62.384				
	*****P*-value**	**0.169**	**0.188**	**0.934**	**0.798**	**0.616**	**0.538**				
**OSI (ratio)**	**Non-pregnant**	4.266± 0.282	3.616± 0.726	4.500± 0.291	3.821± 0.387	5.120± 0.480	5.779± 1.763	**4.517 ± 0.340 ^{A}**			
	**Pregnant**	2.455± 0.404	4.002± 0.435	2.819± 0.330	4.176± 0.375	4.370± 0.358	4.478± 0.476	**3.716 ± 0.196 ^{B}**	**0.038**	**0.068**	**0.358**
	LS Mean (Day)	3.360± 0.345	3.809± 0.410	3.659± 0.315	3.998± 0.264	4.745± 0.306	5.128± 0.896				
	*****P*-value**	**0.004**	**0.658**	**0.003**	**0.523**	**0.236**	**0.500**				

** Values are presented as least-squares means (LS means) ± SE. G, main effect of pregnancy group; D, main effect of sampling day; and G × D, pregnancy group-by-day interaction. For variables with a significant G × D interaction, different lowercase letters ($^{a, b, c}$) indicate significant differences among sampling days within the same group, whereas different uppercase letters ($^{A, B}$) indicate significant differences between the non-pregnant and pregnant groups within the same sampling day ($P<0.05$). Means sharing the same letter or without letters do not differ significantly. The P values presented in the **P-value row below each variable represents pairwise comparisons between the non-pregnant and pregnant groups at each sampling day. When the G × D interaction was not significant, letters accompanying the overall LS means for group or day indicate significant main-effect differences ($P<0.05$). Abbreviations: TOS, total oxidant status; TAS, total antioxidant status; OSI, oxidative stress index; H_2O_2 Equiv., hydrogen peroxide equivalents; Trolox Equiv., Trolox equivalents.*

Table S6. Longitudinal changes in serum progesterone (P_4) and pregnancy-associated glycoprotein (PAG) concentrations in pregnant and non-pregnant ewes from D0 to D50.

Parameter	Group	Day						*LS Mean (Group)*	***P*-value**		
		**D0**	**D7**	**D14**	**D21**	**D35**	**D50**		**G**	**D**	**G × D**
**P_4 (ng/dL)**	**Non-pregnant**	0.148 ± 0.016 ^{b}	0.991 ± 0.183 $^{b, B}$	2.594 ± 0.225 $^{a, B}$	0.151 ± 0.015 $^{b, B}$	0.198 ± 0.021 $^{b, B}$	0.287 ± 0.073 ^{b,B}	**0.728 ± 0.092**	**<0.001**	**<0.001**	**<0.001**
	**Pregnant**	0.212 ± 0.022 ^{e}	3.534 ± 0.339 $^{d, A}$	5.476 ± 0.555 $^{bc, A}$	7.115 ± 0.554 $^{a, A}$	5.939 ± 0.477 $^{ab, A}$	4.471 ± 0.290 $^{cd, A}$	**4.447 ± 0.255**			
	LS Mean (Day)	**0.180 ± 0.014**	**2.263 ± 0.272**	**4.035 ± 0.369**	**3.633 ± 0.598**	**3.002 ± 0.499**	**2.379 ± 0.352**				
	****P-value**	**0.025**	**<0.001**	**<0.001**	**<0.001**	**<0.001**	**<0.001**				
**PAG (O.D.)**	**Non-pregnant**	-	-	-	0.324 ± 0.033 $^{a, B}$	0.197 ± 0.012 $^{b, B}$	0.241 ± 0.060 $^{ab, B}$	**0.254 ± 0.024**	**<0.001**	**<0.001**	**<0.001**
	**Pregnant**	-	-	-	0.491 ± 0.035 $^{c, A}$	1.752 ± 0.078 $^{b, A}$	2.524 ± 0.178 $^{a, A}$	**1.589 ± 0.123**			
	LS Mean (Day)	-	-	-	**0.407 ± 0.027**	**0.974 ± 0.125**	**1.383 ± 0.197**				
	****P-value**				**0.001**	**<0.001**	**<0.001**				

**Values are presented as least-squares means (LS means) ± SE. G, main effect of pregnancy group; D, main effect of sampling day; and G × D, pregnancy group-by-day interaction. For variables with a significant G × D interaction, different lowercase letters ($^{a, b, c}$) indicate significant differences among sampling days within the same group, whereas different uppercase letters ($^{A, B}$) indicate significant differences between the non-pregnant and pregnant groups within the same sampling day (P<0.05). Means sharing the same letter or without letters do not differ significantly. The P values presented in the **P-value row below each variable represents pairwise comparisons between the non-pregnant and pregnant groups at each sampling day. When the G × D interaction was not significant, letters accompanying the overall LS means for group or day indicate significant main-effect differences (P<0.05). PAG was measured only on D21, D35, and D50; "-" indicates that no measurement was performed at the corresponding sampling day. Abbreviations: P_4, progesterone; PAG, pregnancy-associated glycoprotein.*

Table S7. Analysers, reagent kits, analytical principles, and manufacturer-reported performance characteristics for directly measured serum metabolic-profile parameters.

Parameters	Analyser	Reagent Ref./Cat. No.	Analytical principle	Analytical range	Sensitivity / LoD-LoQ	Precision criterion
**Albumin**	AU480	OSR6102	Bromocresol green photometric assay; 600/800 nm	1.5-6.0 g/dL	LoD/LoQ ≤1.5 g/dL	Within-run CV ≤3%; within-laboratory total CV ≤3%
**BUN**	UniCel DxC 800	REF 442750	Urease-GLDH enzymatic rate method; 340 nm	5-100 mg/dL	Sensitivity 5 mg/dL	Within-run CV ≤3%; total CV ≤4.5%
**Ca**	UniCel DxC 800	REF A28937	Indirect potentiometry with calcium ion-selective electrode	2.0-20.0 mg/dL	Sensitivity 2.0 mg/dL	Within-run CV ≤2%; total CV ≤3%
**Cholesterol**	AU480	OSR6116	Enzymatic colorimetric CHE-CHO-POD method; 540/600 nm	20-700 mg/dL	LoD/LoQ ≤20 mg/dL	Within-run CV ≤3%; within-laboratory total CV ≤3%
**CRP**	IMMAGE	REF 447280	Rate nephelometry (antigen-antibody complex formation)	0.1-96 mg/dL	Sensitivity 0.1 mg/dL	Within-run: SD ≤0.05 mg/dL or CV ≤5%; total: SD ≤0.07 mg/dL or CV ≤6.5%
**Glucose**	AU480	OSR6121	Hexokinase/G6P-DH enzymatic UV assay; 340 nm	10-810 mg/dL	LoD/LoQ ≤10.8 mg/dL (serum)	Within-run CV ≤3%; within-laboratory total CV ≤3%
**HDL-C**	AU480	OSR6187	Direct homogeneous enzymatic/chromogenic HDL-C assay	2-180 mg/dL	LoD/LoQ ≤2 mg/dL	Within-run CV ≤3%; within-laboratory total CV ≤4%
**LDH**	AU480	OSR6126	Pyruvate-to-lactate reaction with NADH oxidation; 340 nm	50-3000 U/L	LoD/LoQ ≤50 U/L	Within-run CV ≤5%; within-laboratory total CV ≤10%
**LDL-C**	AU480	OSR6183	Direct enzymatic LDL-C assay (selective protection/deprotection; CHO/PAP)	10-400 mg/dL	LoD/LoQ ≤10 mg/dL	Within-run CV ≤3%; within-laboratory total CV ≤4%
**Mg**	UniCel DxC 800	REF 445360	Calmagite timed-endpoint colorimetric method; 520 nm	0.1-7.0 mg/dL	Sensitivity 0.1 mg/dL	Within-run CV ≤2.5%; total CV ≤3.8%
**P**	UniCel DxC 800	REF 467868	Phosphomolybdate timed-rate method; 365 nm	0.5-12.0 mg/dL	Sensitivity 0.5 mg/dL	Within-run CV ≤2%; total CV ≤3%
**TP**	AU480	OSR6132	Biuret photometric assay; 540/660 nm	3.0-12.0 g/dL	LoD/LoQ ≤3.0 g/dL	Within-run CV ≤3%; within-laboratory total CV ≤4%
**Triglyceride**	AU480	OSR60118	Coupled enzymatic colorimetric assay; 660/800 nm	10-1000 mg/dL	LoD/LoQ ≤10 mg/dL	Within-run CV ≤3%; within-laboratory total CV ≤5%
**Urea**	AU480	OSR6134	Urease-GLDH kinetic UV assay	5-300 mg/dL	LoD/LoQ ≤5 mg/dL	Within-run CV ≤3%; within-laboratory total CV ≤5%

**Analytical ranges, sensitivity or limits of detection/quantification, and precision criteria were obtained from the manufacturers' instructions. Variables reported elsewhere in the manuscript but not included in this table were automatically calculated and reported by the analyser from the directly measured parameters using the following formulas: globulin = TP - albumin; A/G = albumin/globulin; VLDL-C = triglycerides/5; and Ca/P = calcium/phosphorus. Abbreviations: A/G, albumin-to-globulin ratio; BUN, blood urea nitrogen; Ca, calcium; CHE, cholesterol esterase; CHO, cholesterol oxidase; CRP, C-reactive protein; CV, coefficient of variation; G6P-DH, glucose-6-phosphate dehydrogenase; GLDH, glutamate dehydrogenase; HDL-C, high-density lipoprotein cholesterol; LDH, lactate dehydrogenase; LDL-C, low-density lipoprotein cholesterol; LoD, limit of detection; LoQ, limit of quantification; Mg, magnesium; NADH, reduced nicotinamide adenine dinucleotide; P, phosphorus; PAP, phenol-4-aminoantipyrine reaction; POD, peroxidase; SD, standard deviation; TP, total protein; VLDL-C, very-low-density lipoprotein cholesterol.*
